# Facilitators and barriers to facility-based delivery in low- and middle-income countries: a qualitative evidence synthesis

**DOI:** 10.1186/1742-4755-11-71

**Published:** 2014-09-19

**Authors:** Meghan A Bohren, Erin C Hunter, Heather M Munthe-Kaas, João Paulo Souza, Joshua P Vogel, A Metin Gülmezoglu

**Affiliations:** Johns Hopkins Bloomberg School of Public Health, 615 N. Wolfe Street, Baltimore, MD 21205 USA; Department of Reproductive Health and Research, World Health Organization, UNDP/UNFPA/UNICEF/WHO/World Bank Special Programme of Research, Development and Research Training in Human Reproduction (HRP), Avenue Appia 20, Geneva, 1201 Switzerland; The Norwegian Knowledge Centre for the Health Services, Pilestredet Park 7, Oslo, Norway; Department of Social Medicine, Ribeirao Preto School of Medicine, University of Sao Paulo, Ribeirao Preto, Sao Paulo, Brazil

**Keywords:** Maternal health, Obstetric delivery, Obstetric services, Facility-based delivery, Quality of care, Qualitative evidence synthesis, Qualitative systematic review, Disrespect and abuse, Obstetric violence

## Abstract

High-quality obstetric delivery in a health facility reduces maternal and perinatal morbidity and mortality. This systematic review synthesizes qualitative evidence related to the facilitators and barriers to delivering at health facilities in low- and middle-income countries. We aim to provide a useful framework for better understanding how various factors influence the decision-making process and the ultimate location of delivery at a facility or elsewhere. We conducted a qualitative evidence synthesis using a thematic analysis. Searches were conducted in PubMed, CINAHL and gray literature databases. Study quality was evaluated using the CASP checklist. The confidence in the findings was assessed using the CERQual method. Thirty-four studies from 17 countries were included. Findings were organized under four broad themes: (1) perceptions of pregnancy and childbirth; (2) influence of sociocultural context and care experiences; (3) resource availability and access; (4) perceptions of quality of care. Key barriers to facility-based delivery include traditional and familial influences, distance to the facility, cost of delivery, and low perceived quality of care and fear of discrimination during facility-based delivery. The emphasis placed on increasing facility-based deliveries by public health entities has led women and their families to believe that childbirth has become medicalized and dehumanized. When faced with the prospect of facility birth, women in low- and middle-income countries may fear various undesirable procedures, and may prefer to deliver at home with a traditional birth attendant. Given the abundant reports of disrespectful and abusive obstetric care highlighted by this synthesis, future research should focus on achieving respectful, non-abusive, and high-quality obstetric care for all women. Funding for this project was provided by The United States Agency for International Development (USAID) and the UNDP/UNFPA/UNICEF/WHO/World Bank Special Programme of Research, Development and Research Training in Human Reproduction, Department of Reproductive Health and Research, World Health Organization.

**Electronic supplementary material:**

The online version of this article (doi:10.1186/1742-4755-11-71) contains supplementary material, which is available to authorized users.

## Background

Globally, an estimated 287,000 maternal deaths occurred in 2010, with sub-Saharan Africa and South Asia accounting for nearly 85% of the global burden [1]. Evidence-based clinical and preventative interventions aimed at reducing maternal and neonatal morbidity and mortality are well documented [2–5]. One such intervention is increasing skilled attendance at facility-based deliveries [6–8]. According to UNICEF’s 2014 estimates, facility-based delivery rates remain disappointingly low in several regions, including 48% in sub-Saharan Africa, 44% in South Asia, and 71% in the Middle East and North Africa [9]. In the least developed countries, facility-based delivery rates in 2014 averaged 43% [9].

While population-based surveys capture important information regarding the proportion of births occurring in health facilities, surveys are unable to capture the complex explanations for women’s health practices and preferences in delivery location. Qualitative research methods are therefore useful complements to population-based surveys to understand how women perceive, interpret, and weigh a range of factors that affect their delivery location. Synthesizing qualitative evidence allows us to aggregate explanations of the “how” and the “why” behind the decision-making process and the ultimate location of delivery at a facility or elsewhere across multiple contexts. Approaching qualitative evidence synthesis using systematic methodologies increases the transparency, credibility, trustworthiness, and confidence in each of the review findings.

Previous reviews have assessed the health effects of planned hospital birth compared to planned home-birth in low-risk women [10]. Others have identified marginalized womens’ barriers to accessing antenatal care (ANC) in developed countries [11]. This review fills a gap in the literature by systematically synthesizing qualitative evidence related to women’s perceived facilitators and barriers to accessing facility-based deliveries in low- and middle-income countries (LMICs). For the purpose of this review, we have defined a facility-based delivery as a birth occurring in health facility of any level from community health center through tertiary facility. We seek to provide a useful framework for understanding how perceived facilitators and barriers may influence delivery location.

## Methods

### Search strategy

We developed systematic searches for PubMed (Additional file 1: Appendix A) and CINAHL (Additional file 1: Appendix B) using controlled vocabulary and free-text terms combing three components: (a) maternal health, perinatal health, and facility-based delivery; (b) LMICs; and (c) qualitative research methodologies. Searches were conducted in December 2012 and updated in April 2013, with no date limitations. We searched WHO Global Health Library, Cochrane Library, DARE, Google Scholar, CRD, OpenGrey, and EThOs for gray literature and unpublished reports. We also personally contacted researchers in relevant fields of study for assistance in identifying studies. The reference lists of all included studies were hand searched to identify any potentially relevant studies.

### Study selection

The original PubMed and CINAHL search yielded 2,275 articles, from which 101 duplicates were excluded. Three reviewers (MB, EH, HMK) independently screened titles and abstracts for inclusion, then reviewed the full text articles using standardized inclusion criteria: (a) analysis of primary data; (b) English or French language; (c) LMIC; (d) study objectives related to barriers and/or facilitators to facility-based delivery; (e) qualitative data collection method; (f) qualitative analysis method; and (g) full text available. Studies that did not report qualitative data in their findings sections were excluded.

### Quality assessment

MB and EH assessed the quality of included studies using an adaptation of the Critical Appraisal Skills Programme (CASP) quality-assessment tool for qualitative studies (http://www.casp-uk.net/#!casp-tools-checklists/c18f8). The CASP checklist was adapted from a checklist form to a spreadsheet form that allowed for a more in-depth discussion of potential methodological challenges in the primary studies. The modified forms included the following domains: research aims, methodology, research design, recruitment strategy, data collection, data analysis, reflexivity, ethical considerations, findings, and value of research. The overall quality assessment of “high”, “medium”, or “low” was based on the evaluation by two reviewers and active discussion until consensus was reached in the case of rating discrepancies. No studies were excluded as a result of the quality assessment; rather, the methodological rigor of each contributing study contributed to the confidence assessments of each review finding.

### Data extraction

MB and EH used a standardized form to extract data pertaining to the following domains: study setting and demographics, study objectives, study design, data collection and analysis methods, themes, and conclusions. MB contacted and received further information from four authors concerning their data collection and analysis methods when the published studies lacked sufficient detail.

### Synthesis

MB and EH created a spreadsheet of all relevant data extracted from the included studies’ findings sections and used thematic analysis methods to conduct initial open coding on each relevant text unit [12]. The initial round of coding developed the themes presented in Table 1. All text units were subsequently classified into one of the themes in an iterative manner. The initial coding scheme was intentionally very broad in order to capture the overarching core themes present in the data. Then, each theme was further analyzed to develop the axial coding scheme (Additional file 1: Appendix C). Axial coding is widely accepted in qualitative literature as a sufficient method to disaggregate core themes during qualitative analysis [13, 14]. MB and EH applied the axial codes systematically to the data by hand-sorting the text units into themes and sub-themes. Table 2 pictographically presents the first, second, and third order themes that emerged from the initial and axial coding. First order themes represent text units that are grouped together based on common themes. Second order themes represent first order themes grouped together based on common, higher-level themes. Third order themes represent overarching high-level themes comprised of the first- and second-level themes [15].

**Table 1:** **Analytic framework**

Theme	Description
Cost	Direct and indirect costs associated with facility birth
Influence of others on birthing decisions	Involvement of husbands, partners, family members, and friends on delivery location decisions
Plan for childbirth	Plans or lack of plans that a woman or her family make for her delivery
HIV	Fear of HIV testing, disclosure, and discrimination
Transportation/access	Perception of the distance and time to a health facility and implications of available transportation options.
Policies	Health policies that may influence the decision to deliver in a facility or at home
Perception of risk	Awareness of risks associated with childbirth, influence of previous birth experiences on future delivery choices, and influence of ANC on delivery choice.
Perceived quality of care	Perceived quality of care received at facilities during delivery
Medicalization of childbirth	The perception that birth is a natural event, lack of supportive attendance at facility deliveries, fear of cutting
Intersection of traditionalism and modernity	Influence of tradition and culture on delivery decisions, delays in transition from unskilled to skilled care, cooperation between traditional and biomedical health systems
Logistics of home birth	Perception that home deliveries are logistically easier than facility deliveries

Initial framework and themes for analysis. Each theme was broken down into sub-themes in the second round of analysis. See appendix C for full codebook.

**Table 2:** **Thematic analysis**

Third order	Second order	First order	References
**Perceptions of pregnancy and delivery**	**Traditional influences**	Barrier: Tradition supports an external locus of control	[16–20]
		Barrier: Traditional understandings of disease etiology	[16, 19–23]
	**Medicalization of childbirth**	Barrier: Facilities deemed unnecessary for the “natural event” of birth	[18, 19, 24–37]
		Facilitator: Facility delivery valued for obstetric complications	[18, 19, 26, 29–38]
		Barrier: Unfamiliar and undesirable birth practices in facilities	[18, 19, 22, 24, 26, 29–31, 36, 39, 40]
		Barrier: Lack of privacy in a facility	[24, 26, 27, 31, 39, 41, 42]
		Barrier: Lack of supportive attendance during facility delivery	[23, 24, 29, 34, 36, 39, 41, 43]
		Barrier: Fear of cutting	[19, 22–24, 36, 43–45]
		Facilitator: Desire for modernity	[16, 18, 20, 24, 25]
		Barrier: Making logistical plans for childbirth is rare	[18, 19, 25, 29, 31, 32]
**Influence of sociocultural context and care experiences**	**Influence of ANC**	Barrier: Belief that ANC diminishes the likelihood of a complicated delivery	[19, 30, 46]
		Barrier: ANC providers do not universally promote facility delivery	[19, 29, 35, 45]
		Barrier: Lack of ANC attendance inhibits facility delivery	[27, 28, 31]
	**Previous birth experiences**	Facilitator/barrier: Effects of previous birth experiences on subsequent delivery locations	[17, 20, 21, 24, 25, 30, 32, 34, 36, 39, 44, 46, 47]
	**Influence of others on delivery location**	Barrier: Too many people involved in the decision-making process leads to delays in seeking care	[16, 18, 23, 24, 26, 29, 32, 33, 36, 40, 45, 47]
		Barrier: Intergenerational continuity and the role of elder women	[16–19, 21, 34, 39, 43, 45]
		Facilitator/barrier: The role of husbands	[16–22, 24, 25, 28, 29, 31, 39, 41, 47]
		Facilitator: Personal links to healthcare facilities	[20, 25, 32, 44]
	**Ease of home birth**	Barrier: Facility births less convenient than home births	[18, 33, 35, 42, 46]
		Barrier: Unable to maintain household or family demands during facility delivery	[18, 19, 21, 32, 33, 45]
	**Effects of policies**	Facilitator/barrier: Health insurance schemes, national population policies, and national policies aimed to shift deliveries from the home to a facility	[21, 23, 31, 33, 34, 46, 48]
**Resource availability and access**	**Transportation**	Barrier: Poor proximity and access to a facility	[18, 20, 25–27, 29, 32–36, 39, 41, 45, 46, 49]
		Barrier: Lack of accessible and reliable transportation	[25–27, 32, 36, 39, 41, 45, 47]
		Barrier: Inaccessibility of transportation and facilities during off-hours	[33, 39, 41, 43, 45, 46]
		Barrier: Delays in accessing referral services	[17, 29, 34, 45, 49]
	**Cost of childbirth**	Barrier: Perceived high cost of facility birth compared to home birth	[17, 20, 23, 24, 26, 28, 30, 32–37, 39, 41, 42, 46, 48, 49]
		Barrier: Lack of access to funds in an emergency	[28, 32–34, 37, 41, 48, 49]
		Barrier: Indirect and hidden costs associated with facility delivery	[16, 19, 20, 24, 25, 30, 31, 34, 41, 43–45, 47–49]
**Perceptions of quality of care**	**Perceived quality of care from TBAs**	Barrier: Utilization of TBAs as first-line providers	[18, 23, 24, 30, 32, 33, 37, 39, 40, 43–46]
		Facilitator: TBAs perceived as providing low quality care	[20, 30, 33, 39, 43, 45]
		Barrier: TBAs perceived as providing high quality care	[18, 19, 21, 22, 30, 33, 35, 40, 45]
	**Perceived quality of care at facilities**	Facilitator: Facilities perceived as providing high quality care	[16–19, 21, 24, 29, 30, 33–35, 39, 41, 45, 47]
		Barrier: Facilities perceived as providing low quality of care	[17–19, 23, 26, 29–31, 36, 39]
		Barrier: Mistreatment and abuse by health workers	[17–21, 24, 28, 34, 36, 37, 41, 42, 45–48]
		Barrier: Neglect and delays in receiving care at the facility	[17, 24, 31, 36, 39, 41, 45, 46, 48]
		Barrier: Inadequate health facility staffing and infrastructure	[17, 18, 24, 34, 35, 37, 39, 41, 45–47]
	**Stigma**	Barrier: Fear of compulsory HIV testing during delivery services	[28, 30, 36, 38]
		Barrier: Fear of HIV-status disclosure in health facilities	[28, 36, 38]
		Barrier: Fear of treatment disparities among HIV-positive women	[28, 38]
		Barrier: Stigmatization of unwed, pregnant women	[17, 21, 41]

### Assessing the confidence of the findings

MB and EH assessed the confidence of each review finding using the first version of the CERQual (Confidence in the Evidence from Reviews of Qualitative Research) tool. The CERQual tool is designed to assess the reviewer’s confidence in each individual review finding, and is not a methodological quality appraisal tool. The CERQual tool is under development and the version of CERQual that we used includes two elements, methodological quality and coherence (the current version of CERQual is comprised of four components) [50–55]. First, we appraised the methodological quality of the individual studies contributing to each review finding using the modified CASP tool discussed previously. The methodological assessment of the individual studies contributing to each review finding is important to determine how likely it is that the research produced credible results, how precise and dependable an understanding of the phenomenon of interest the research will provide, and how widely the research findings could be applied. In the CERQual approach, confidence in a review finding is weakened when the primary studies that contribute to each review finding have critical methodological weaknesses. Second, we assessed the coherence of each review finding by exploring to what extent clear patterns could be identified across the data contributed by each of the individual studies, or that plausible explanations are provided if there is variation across individual studies. Assessing coherence of each review finding is important as it encourages the reviewers to examine whether each review finding is well grounded in data from the primary studies. The main threat to the coherence of a review finding is unexplained inconsistencies found from variations in the data from individual studies. Based on the assessment of the methodological quality of individual studies contributing to each review finding and the coherence of each review finding, the confidence in the evidence for each review finding was assessed as high, moderate, and low (Table 3).

**Table 3:** **Summary of findings**

#	Factors that affect the utilization of facility-based deliveries	Relevant papers	Confidence in the evidence	Explanation of confidence in the evidence assessment
1	**Barrier: Tradition supports an external locus of control**	[16–20]	Moderate confidence	In general, the studies were moderately well done. The finding was seen across several studies and settings.
	Across sub-Saharan Africa, religious faith and traditional religious practices played a role in decision-making regarding delivery location. Women described their trust in God and the belief that God controls their destiny. These traditional beliefs contributed to a sense of fatalism as some women believed that delivery complications were beyond their control.			
2	**Barrier: Traditional understandings of disease etiology**	[16, 19–23]	Moderate confidence	In general, the studies were moderately well done. The finding was seen across several studies and settings.
	Seeking care at medical facilities may have been delayed in situations when women or their families viewed certain health problems as spiritual rather than physical in nature, influenced by their traditional understandings of disease etiologies.			
3	**Barrier: Facilities deemed unnecessary for the “natural event” of birth**	[18, 19, 24–37]	High confidence	In general, the studies were moderately well done. The finding was seen across many studies and settings.
	The perception that birth is a natural life event rather than a medical procedure emerged as a common theme in many of the primary studies across a variety of contexts. Respondents therefore saw no rationale for delivering at a facility, and paying to do so was considered illogical and superfluous.			
4	**Facilitator: Facility delivery valued for obstetric complications**	[18, 19, 26, 29–38]	High confidence	In general, the studies were moderately well done. The finding was seen across many studies and settings.
	Many women across different contexts attempted home delivery first and considered facilities acceptable only if complications arose during the delivery process. Although facility-based delivery was not the first choice for many women, they acknowledged the importance of facilities in cases of complicated birth.			
5	**Barrier: Unfamiliar and undesirable birth practices in facilities**	[18, 19, 22, 24, 26, 29–31, 36, 39, 40]	High confidence	In general, the studies were moderately well done. The finding was seen across many studies and settings.
	When faced with the prospect of facility birth, some women may fear unfamiliar or undesirable procedures, such as unfamiliar birthing positions and intrusive vaginal exams. Hospital providers were sometimes perceived to conduct too many digital vaginal examinations, which women found uncomfortable and dehumanizing. Some women also preferred delivering at home with a TBA because they had more control over their birth position than delivering at a facility.			
6	**Barrier: Lack of privacy in a facility**	[24, 26, 27, 31, 39, 41, 42]	Moderate confidence	In general, studies were moderately well done. The finding was seen across several studies and settings.
	Many women felt that they had more control over maintaining their privacy when delivering at home compared to the facility. Privacy is greatly valued by parturient women, yet it may not be well-maintained in a facility due to a lack of cultural sensitivity and dismissive attitudes towards poor women, coupled with the lack of private labor wards.			
7	**Barrier: Lack of supportive attendance during facility delivery**	[23, 24, 29, 34, 36, 39, 41, 43]	Moderate confidence	In general, studies were moderately well done. The finding was seen across several studies and settings.
	One of the most salient differences between home birth and facility birth was the perceived lack of supportive attendance at birth in a facility. Women commonly referred to their families and TBAs as providing supportive and comforting care, and receiving physical, social, and emotional support from their family during delivery was vitally important for the parturient woman. Facility policies limiting the involvement of TBAs and family members during birth induced anxiety in many women.			
8	**Barrier: Fear of cutting**	[19, 22–24, 36, 43–45]	Moderate confidence	In general, studies were moderately well done. The finding was seen across several studies and settings.
	Across multiple contexts, women referred to a “fear of cutting” as a deterrent to pursuing facility delivery. Women who mentioned a fear of cutting usually did not differentiate between episiotomy and a caesarean section; rather, they referred to any form of perineal or abdominal incision as “cutting”. Women feared cutting due to perceived longer hospital stays, higher cost, perceived unjustified operation, social stigma, and potential problems with future sexual relations.			
9	**Facilitator: Desire for modernity**	[16, 18, 20, 24, 25]	Moderate confidence	In general, studies were moderately well done. However, this finding was only seen in 4 countries.
	Despite the role of tradition in delivery practices, women, husbands, and traditional leaders commented on changing societal norms regarding the location of delivery. In some contexts, women viewed facility delivery as a modern or contemporary idea and as something to which they aspire.			
10	**Barrier: Making logistical plans for childbirth is rare**	[18, 19, 25, 29, 31, 32]	Moderate confidence	In general, studies were moderately well done. The finding was seen across several studies and settings.
	Across several contexts, the lack of planning in advance for childbirth, including the decision about location of delivery, transportation planning, and acquiring liquid assets to pay for associated childbirth costs, prevented women from accessing facility delivery. Families often lack the resources to develop coping mechanisms for future events. Therefore, the capacity to make plans in low-resource households is inherently difficult. Women and their families viewed childbirth as an unpredictable event, which made creating a birth plan difficult.			
11	**Barrier: Belief that ANC diminishes the likelihood of a complicated delivery**	[19, 30, 46]	Low confidence	In general, studies were moderately well done. However, this finding was only present in 3 studies in 3 countries.
	Some women viewed ANC as a means to ensure a normal pregnancy and childbirth and to prepare for *home* delivery. A facility delivery would therefore not be considered unless an ultrasound during an ANC visit suggested that the mother or baby were in danger because ultrasounds are believed to be able to predict whether or not a woman will have an uncomplicated or “normal” delivery. Furthermore, ANC itself was understood by some to actually reduce the risk of complications during delivery, which may help to explain why in some contexts ANC coverage is near universal while facility delivery rates remain low.			
12	**Barrier: ANC providers do not universally promote facility delivery**	[19, 29, 35, 45]	Low confidence	In general, studies were moderately well done. However, this finding was only present in 4 studies in 4 countries.
	ANC providers may not be adequately advising women of the importance of facility-based care during delivery. Providers may also neglect to discuss the importance of planning ahead, instead only suggesting facility-based delivery for women with identifiable danger signs. ANC providers may be unintentionally encouraging home births by providing information on making home-birth safer (i.e. providing advice on safe home-based cord cutting measures), thus validating the practice.			
13	**Barrier: Lack of ANC attendance inhibits facility delivery**	[27, 28, 31]	Low confidence	In general, studies were moderately well done. However, this finding was only present in 3 studies in 3 countries.
	Some women may not feel comfortable delivering in a facility if they have not attended ANC, even if they otherwise desire a facility birth. These women may fear mistreatment from heath workers for not possessing an ANC card or may avoid the facility due to poor experiences during ANC care.			
14	**Facilitator and barrier: Effects of previous birth experiences on subsequent delivery location**	[17, 20, 21, 24, 25, 30, 32, 34, 36, 39, 44, 46, 47]	High confidence	In general, studies were moderately well done. Diverse findings were seen across many studies and settings.
	Across a variety of contexts, women determined their level of risk for complicated deliveries based on their prior delivery experiences and birth outcomes, and these previous birth experiences may act as either a facilitator or barrier to future delivery deliveries. In many contexts, a woman’s first delivery is considered the riskiest since she has no prior experiences with child birth. Women who had previous cesarean sections or obstetric complications may desire future facility delivery due to higher perceived risk. However, if a woman gave birth to her first child without complications, utilizing a facility for subsequent births may be viewed as unnecessary or illogical. Likewise, previous negative experiences with facility births may deter women from delivering at a facility during a future birth.			
15	**Barrier: Too many people involved in the decision-making process leads to delays in seeking care**	[16, 18, 23, 24, 26, 29, 32, 33, 36, 40, 45, 47]	High confidence	In general, the studies were moderately well done. The finding was seen across many studies and settings.
	Across many contexts, parturient women may not be in full control of the decision to seek facility-based delivery, instead relying on the decisions made by many actors, including elder women, husbands, family members, and neighbors. These actors may have competing interests in the choice of a woman’s delivery location, and obtaining advice and approval from them often delays or prevents facility delivery, particularly because these decisions are often sought after labor has begun.			
16	**Barrier: Intergenerational continuity and the role of elder women**	[16–19, 21, 34, 39, 43, 45]	High confidence	In general, the studies were moderately well done. The finding was seen across many studies and settings.
	Across a variety of contexts, elder women, including mothers, mothers-in-law and grandmothers of parturient women, hold the greatest influence and decision-making power regarding delivery location. Some women believed that they should choose the same delivery location that their mothers and grandmothers experienced, in order to maintain their identity and intergenerational continuity. Other women may be pressured by the elder women to deliver at home.			
17	**Facilitator and barrier: The role of husbands**	[16–22, 24, 25, 28, 29, 31, 39, 41, 47]	Low confidence	In general, the studies were moderately well done. The role of the husband was seen across many studies and settings. However, the diverse range of roles that husbands play makes it difficult to draw conclusions on whether their role is a facilitating or inhibiting factor in accessing facility delivery.
	The husband plays a complex role in facilitating or preventing his wife from accessing facility-based delivery and this role varies across different contexts. In some settings, a husband may act as a facilitator by persuading his wife to visit a facility and mobilizing the necessary transportation and funds. In contrast, a husband may prohibit a facility visit altogether due to financial or cultural constraints. In other settings, the husband may play a more neutral role and place the decision to seek care in someone else’s hands, such as elder female family members. Although this finding was explored in 15 studies across 9 countries, the role of husbands varied so greatly both within and between study populations that it is difficult to draw any macro-level conclusions other than that the husband plays an important role in deciding where to deliver.			
18	**Facilitator: Personal links to healthcare facilities**	[20, 25, 32, 44]	Low confidence	In general, studies were moderately well done. However, the finding was only from 3 countries.
	Families with social connections to skilled providers may be more accepting of the biomedical approach to maternity care and thus more willing to seek a facility-based delivery. More importantly, a relative or friend working at a nearby facility can often arrange quicker admission or quality treatment of a parturient woman. However, this finding was only seen in 4 studies across 3 countries, including 3 studies in Bangladesh.			
19	**Barrier: Facility births less convenient than home births**	[18, 33, 35, 42, 46]	Moderate confidence	In general, studies were moderately well done. The finding was seen across several studies and settings.
	In several contexts, women preferred to deliver at home, where they were in a familiar and convenient setting. During a homebirth, a woman would not need to arrange for child care or transportation, could rest in her own bed after delivery, and be catered to by her family and friends.			
20	**Barrier: Unable to maintain household or family demands during facility delivery**	[18, 19, 21, 32, 33, 45]	Moderate confidence	In general, studies were moderately well done. The finding was seen across several studies and settings.
	Some women felt that they could exert greater control on their domestic responsibilities when they delivered at home and were concerned that their domestic responsibilities, such as child care, cooking, cleaning, gardening and tending the livestock, would be abandoned if they attended a health facility for delivery.			
21	**Barrier: Poor proximity and access to a facility**	[18, 20, 25–27, 29, 32–36, 39, 41, 45, 46, 49]	High confidence	In general, the studies were moderately well done. The finding was seen across many studies and settings.
	Geographical distance to a health facility is an influential factor affecting a woman’s delivery location, explored in 16 studies across 11 countries. Women residing in both urban and rural areas where health services do not exist at the community level may face considerable traveling time to reach a facility. The perceived far distance to health facilities may create a dependency on home birth as some women report that the facility is too far to travel to during labor, particularly given the restricted transportation options.			
22	**Barrier: Lack of accessible and reliable transportation**	[25–27, 32, 36, 39, 41, 45, 47]	Moderate confidence	In general, studies were moderately well done. The finding was seen across several studies and settings.
	Poor availability of transportation played a crucial role in the decision to deliver at a facility and whether or not it could be reached in a timely manner. In the absence of a reliable private car, women were faced with arduous modes of transportation including bicycle, rickshaw, motorcycle, boat, walking, or public transportation, which was often intermittent in rural areas.			
23	**Barrier: Inaccessibility of transportation and facilities during off-hours**	[33, 39, 41, 43, 45, 46]	Low confidence	In general, studies were of low quality. The finding was seen across several studies and settings.
	Travel at night or on weekends was considered particularly difficult as there are fewer public transportation options, women may be afraid of thieves and wild animals, and the price is higher. Even if women are able to arrange transportation during the off-hours, health facilities may be closed or lack the staffing to manage her delivery.			
24	**Barrier: Delays in accessing referral services**	[17, 29, 34, 45, 49]	Moderate confidence	In general, studies were moderately well done. The finding was seen across several studies and settings.
	Organizing referrals for obstetric complications was a time-consuming and arduous process, complicated by a lack of access to transportation, good roads, adequate funds, and communication systems. The lack of coordination between different health system actors also contributed to delays in reaching care.			
25	**Barrier: Perceived high cost of facility birth compared to home birth**	[17, 20, 23, 24, 26, 28, 30, 32–37, 39, 41, 42, 46, 48, 49]	High confidence	In general, the studies were moderately well done. The finding was seen across many studies and settings.
	Direct costs associated with childbirth were perceived to be unaffordable for many women and some women perceived themselves as too poor to deliver in a facility. Where women viewed childbirth as a non-medical event, the cost of childbirth is considered extraneous and unnecessary. This finding was explored in 19 studies across 12 countries.			
26	**Barrier: Lack of access to funds in an emergency**	[28, 32–34, 37, 41, 48, 49]	Moderate confidence	In general, studies were moderately well done. The finding was seen across several studies and settings.
	Low-SES families often did not plan in advance for costs associated with child birth and few families had assets or savings to devote to health expenses, thus causing a scramble to raise funds during obstetric complications. Collecting the necessary money was a difficult task as few banks or moneylenders would lend money to the poor, and if they did, exorbitant interest rates could make the principle escalate rapidly in just a few months. Instead, family members were often sent around the community to collect money from their neighbors or try to sell property or livestock			
27	**Barrier: Indirect and hidden costs associated with facility delivery**	[16, 19, 20, 24, 25, 30, 31, 34, 41, 43–45, 47–49]	High confidence	In general, the studies were moderately well done. The finding was seen across many studies and settings, but predominantly in Bangladesh and Tanzania.
	Even in settings where direct delivery costs were subsidized, families were expected to pay for transportation to the facility, drugs, medical supplies (i.e.: gloves, needles, gauze), blood for transfusions, laboratory services, food during the hospital stay, bribes to health providers, and laundry services. These additional costs often came as a surprise to women after they attended the facility, which may impact their future choice of delivery location. In addition to the extra point-of-care costs associated with facility birth, families experienced opportunity costs due to absence from work and domestic responsibilities.			
28	**Barrier: Utilization of TBAs as first-line providers**	[18, 23, 24, 30, 32, 33, 37, 39, 40, 43–46]	High confidence	In general, studies were moderately well done. The finding was seen across many studies and settings.
	TBAs played an important role as first-line providers for many women and this role was discussed in 13 studies across 10 countries. Women emphasized the close bond that they felt with TBAs, due to their status in the community and the trust they developed over years of experience. This relationship often prompted women to desire home-based births attended to by a TBA rather than a facility.			
29	**Facilitator: TBAs perceived as providing low quality care**	[20, 30, 33, 39, 43, 45]	Moderate confidence	In general, the studies were moderately well done. The finding was seen across several studies and settings.
	Despite the bond that many women had with TBAs in their community, some women perceived TBAs as providers of low quality delivery care. These women did not trust the TBAs’ skills, knowledge, or ability to handle complications and may be more likely to seek facility-based delivery.			
30	**Barrier: TBAs perceived as providing high quality care**	[18, 19, 21, 22, 30, 33, 35, 40, 45]	Moderate confidence	In general, the studies were moderately well done. The finding was seen across many studies and settings.
	Other women perceived TBAs as providing high quality delivery care, often emphasizing the supportive and emotional role that TBAs play. These women may believe that TBAs have innate skills gifted to them from God and that TBAs are more dependable providers than facility-based health workers.			
**Experiences with facility providers**				
31	**Facilitator: Facilities perceived as providing high quality care**	[16–19, 21, 24, 29, 30, 33–35, 39, 41, 45, 47]	High confidence	In general, the studies were moderately well done. The finding was seen across many studies and settings.
	In contexts where facilities are perceived as providing high quality care, women may seek facility delivery to ensure positive birth outcomes. They may view facilities as providing efficacious and respectable care, and health workers as compassionate experts. It is important to note that within the same study area, perceptions of facility-based care vary greatly and participants more commonly perceived facilities to have low quality of care than high quality of care. However, women who perceived facilities as providing high quality care reportedly felt more comfortable seeking facility-based delivery.			
32	**Barrier: Facilities perceived as providing low quality of care**	[17–19, 23, 26, 29–31, 36, 39]	High confidence	In general, the studies were moderately well done. The finding was seen across many studies and settings.
	Across multiple contexts, the failure of health workers to manage severe obstetric complications contributed to a negative image of facility delivery. Women may lack confidence in the abilities of the health workers, who they consider to be undertrained, lacking skills, incompetent, inexperienced, and offering inaccurate diagnoses. It is important to note that even within the same study area, perceptions of facility-based care vary greatly. However, women who perceived facilities as providing low quality care reportedly felt less likely to seek facility-based delivery.			
33	**Barrier: Mistreatment and abuse by health workers**	[17–21, 24, 28, 34, 36, 37, 41, 42, 45–48]	High confidence	In general, the studies were moderately well done. The finding was seen across many studies and settings.
	Many women referred to poor patient-provider interactions as a barrier to seeking delivery care. Women described providers as verbally abusive, rude, bossy, unhelpful, disrespectful, critical, easily angered, having a poor attitude, and lacking compassion. Respondents reported that facility-based providers shout at, physically abuse, and insult women during delivery.			
34	**Barrier: Neglect and delays in receiving care at the facility**	[17, 24, 31, 36, 39, 41, 45, 46, 48]	Moderate confidence	In general, the studies were moderately well done. The finding was seen across several studies and settings.
	Upon arrival to a facility, women often experienced delays in care provision and health workers were often slow to respond to patient needs. Health workers often did not communicate with the woman or her family on the progress of labor.			
35	**Barrier: Inadequate health facility staffing and infrastructure**	[17, 18, 24, 34, 35, 37, 39, 41, 45–47]	Moderate confidence	In general, the studies were moderately well done. The finding was seen across many studies and settings.
	Inadequate staffing and infrastructure in the facilities contributed to the perceived low quality of care. The lack of adequate staffing led to overburdened lower-level providers and often prompted women to visit untrained traditional providers to respond to the gaps in service.			
**Experiences with stigmatization in facilities**				
36	**Barrier: Fear of compulsory HIV testing during delivery services**	[28, 30, 36, 38]	Low confidence	In general, the studies were moderately well done. However, the finding was only from 4 studies in Kenya. Therefore, the confidence of the finding across multiple contexts is low, but may be higher in Kenya.
	In high HIV prevalence settings, a fear of compulsory HIV testing during facility-based delivery sometimes prompted women to avoid facilities altogether. These women feared the shock, stress, and depression caused by a positive HIV test, often believing that knowledge of one’s own positive HIV-status was as equally deleterious as the virus itself. This finding was present in 4 studies in 1 country (Kenya), so the certainty of the finding across multiple contexts is low, but may be higher in the Kenyan context.			
37	**Barrier: Fear of HIV-status disclosure in health facilities**	[28, 36, 38]	Low confidence	In general, the studies were moderately well done. However, the finding was only from 3 studies in Kenya and may only be applicable to high HIV prevalence settings.
	Women feared unwanted disclosure of their positive HIV-status in a facility, which could lead to tremendous social, psychological, physical, and economic consequences. Crowded maternity wards, public administration of ARVs, and health workers’ failure to maintain strict confidentiality sometimes caused women to avoid facility deliveries. Again, this finding was present in 3 studies in 1 country (Kenya), so the certainty of the finding across multiple contexts is low, but may be higher in the Kenyan context.			
38	**Barrier: Fear of treatment disparities among HIV-positive women**	[28, 38]	Low confidence	In general, the studies were moderately well done. However, the finding was only from 2 studies in Kenya.
	Some HIV-positive women may be provided with lower quality of care due to health workers’ fear of HIV infection. However, this finding was only present in 2 studies in 1 country (Kenya).			
39	**Barrier: Stigmatization of unwed, pregnant women**	[17, 21, 41]	Low confidence	In general, the studies were moderately well done. However, the finding was only from 3 studies in Sierra Leone, Tanzania, and Vietnam.
	Most societies view pregnancy and childbirth as the outcome of a marital relationship, thereby potentially stigmatizing and disempowering unwed women seeking facility delivery. Delivering at home was a desirable choice for unwed women or adolescents to avoid embarrassment or discrimination at a facility, particularly because these women were often lacking emotional and financial support from their partner or parents. However, this finding was only present in 3 studies in 3 countries (Sierra Leone, Tanzania, and Vietnam).			

### Role of the funding source

The funder of this review had no role in the study design, analysis, or writing of the report. Funding for this project was provided by The United States Agency for International Development (USAID) and the UNDP/UNFPA/UNICEF/WHO/World Bank Special Programme of Research, Development and Research Training in Human Reproduction, Department of Reproductive Health and Research, World Health Organization.

### Reporting

This systematic review is reported following the ENTREQ statement guidelines to enhance transparency in reporting qualitative evidence synthesis [56].

### Findings

**Figure 1:**
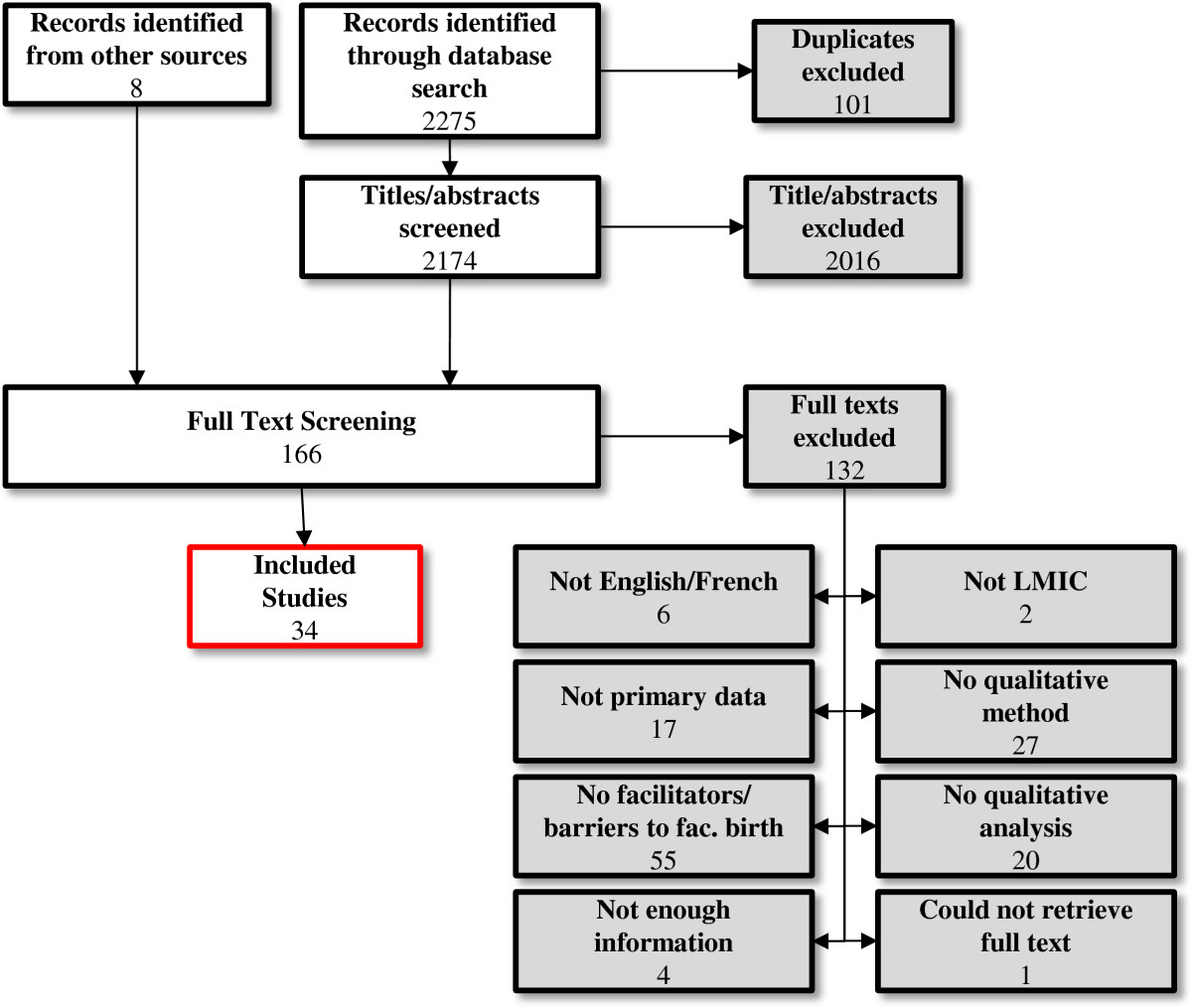
**Flow diagram of search and inclusion process.**

**Figure 2:**
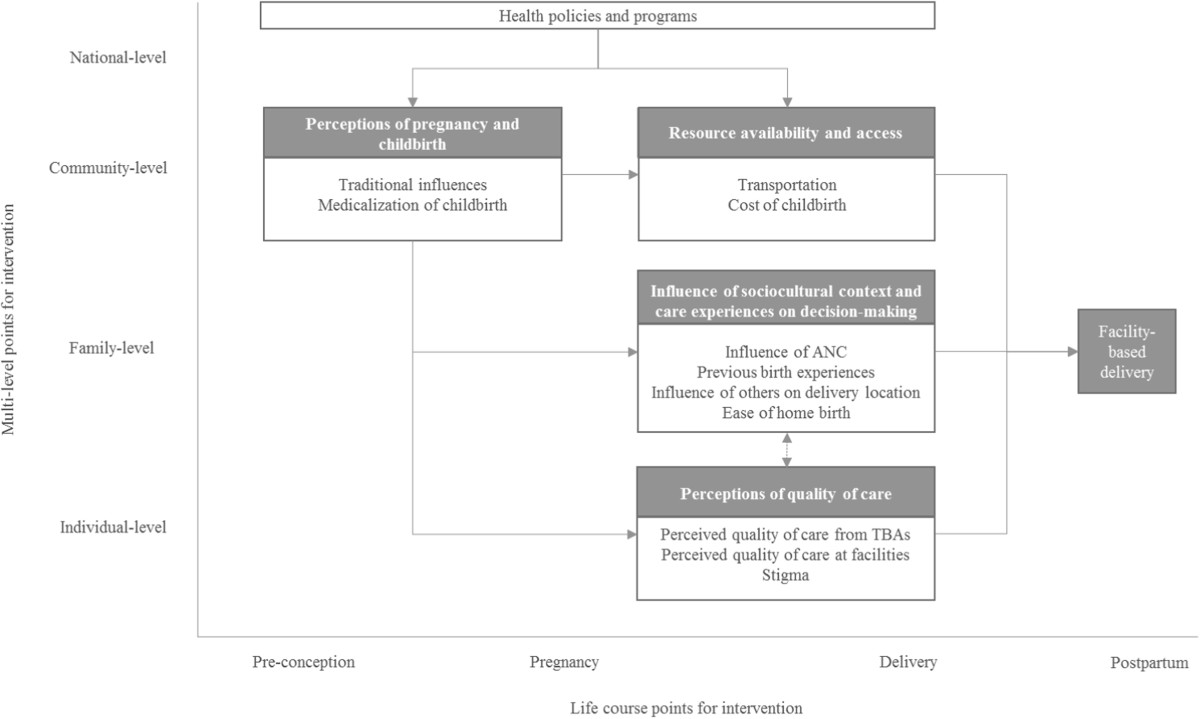
**Multi-level life course framework of facility-based delivery in LMICs.** This framework was developed using the multi-level life course approach to explore how experiences earlier in an individual’s life impact their subsequent decisions and actions, and how these experiences range across individual, family, community, and national level influences. The framework was developed after the review findings and first, second, and third order themes were finalized.

A total of 34 studies were included from 17 LMICs in Africa (8 countries), Asia (7 countries), South America, (1 country) and the Middle East (1 country). Figure 1 presents the review’s flow diagram. Study summaries are presented in Additional file 1: Appendix D. First-order descriptive themes and second- and third-order analytic themes are summarized in Table 2 and discussed in the following sections. Figure 2 presents a multilevel life course conceptual framework of accessing facility-based delivery. The complete summary of findings and corresponding confidence assessments are in Table 3.

### Perceptions of pregnancy and delivery

#### Traditional influences

Traditional influences including local understandings of disease etiology and externally-focused loci of control play complex but important roles in understanding decision-making on location of delivery [16–22, 41]. Care-seeking may be delayed in situations where certain health problems are viewed as spiritual in nature rather than physical, such as eclamptic seizures [16, 19–23]. Despite the role of tradition in delivery practices, several respondents referred to home birth as “old time” and desired the modernity of facility-based delivery [16, 18, 20, 24, 25].

### Medicalization of childbirth

Both women and men described the birthing process as a “normal” or “routine” event and believed that childbirth was a woman’s “natural rite of passage” [18, 19, 24–37]. Therefore, there was no rationale for delivering at a facility, and paying to do so was considered illogical and superfluous. Many women attempted home delivery first and considered facility birth only if complications arose [18, 19, 26, 29–38].

When faced with the prospect of facility birth, women feared undesirable birth practices, such as unfamiliar birthing positions [18, 19, 22, 24, 26, 29–31, 36, 39, 40]. They preferred delivering at home with TBAs to retain control over their birth position. Medicalization of childbirth can leave women with the feeling that they are no longer active participants or decision-makers in the birthing process [24]. Hospital providers were perceived as conducting unnecessary vaginal examinations, which women found uncomfortable and dehumanizing [19, 24]. Women viewed childbirth as an unpredictable event, which made creating a birth plan difficult [19, 25, 31]. This lack of planning in advance for childbirth, including decisions regarding delivery location, transportation, and availability of cash, prevent many women from accessing facility delivery [18, 19, 25, 29, 31, 32].

Many women felt more in control of maintaining their privacy when delivering at home [24, 26, 27, 31, 39, 41, 42]. Privacy is greatly valued by parturient women, yet it may be difficult to achieve in a facility due to cultural insensitivity, [24] or a lack of private labor wards [26, 27, 42]. The lack of supportive attendance during facility-based delivery was a major concern [23, 24, 29, 34, 36, 39, 41, 43]. Women commonly referred to their families and TBAs as providing supportive care during home births.

The “fear of cutting” (episiotomy or caesarean section) during delivery is an important barrier to facility-based delivery [19, 22–24, 36, 43–45]. Since many women believe that “a woman is born to deliver vaginally,” [35] caesarean sections are seen as an unnatural intervention. Caesarean sections are also believed to be used indiscriminately without thorough consideration regarding individual cases [25, 34, 51, 54]. Similarly, women viewed episiotomy as an unnecessary intervention with complex social impacts [17, 19, 26].

### Influence of sociocultural context and care experiences

#### Influence of antenatal care

Women may believe that attending ANC will diminish the likelihood of a complicated delivery, and use ANC in a preventative manner as a means to ensure a normal pregnancy and home-birth [19, 30, 46]. This may explain why in some contexts ANC coverage is near universal while facility delivery rates remain low [19, 30, 46]. In settings where ANC attendance was nearly universal, those few women who did not seek ANC felt uncomfortable seeking facility-based delivery due to their unfamiliarity with the health system and fear of mistreatment for not possessing an ANC attendance card [27, 28, 31]. ANC providers may not be adequately advising women of the importance of facility-based delivery [19, 29, 35, 45] due to a heavy workload and limited time to discuss complex issues with their patients [19]. Some providers hesitate to encourage all women to deliver at a facility because of the scarcity of space or equipment [45].

### Previous birth experiences

Women determine their level of risk for complicated deliveries in part based on their prior delivery experiences and birth outcomes, which informs their future delivery location. A woman may be more likely to deliver at a facility during her first birth [34] or if she had a previous obstetric complication [25, 32, 34, 44]. However, if a woman delivered her first child without complications, utilizing a facility for subsequent births is often viewed as unnecessary [20, 21, 24, 30, 34, 36, 39, 46, 47].

### Influence of others on delivery location

A parturient woman may not be in control of the decision to seek facility-based delivery, instead relying on decisions made by elder women, husbands, other family members, and neighbors [16–26, 28, 29, 31–34, 36, 39–41, 43–45, 47]. While the influence of some actors may facilitate accessing skilled care, the involvement of too many actors often results in the delay or prevention of facility-based births [16, 18, 23, 24, 26, 29, 32, 33, 36, 40, 45, 47].

Elder women hold the greatest influence and decision-making power regarding delivery location across Asia and sub-Saharan Africa [16–19, 21, 34, 39, 43, 45]. Some women believed that they should choose the same delivery location as their mothers and grandmothers to maintain intergenerational continuity, and elder women may pressure younger women to deliver at home [18, 21, 34, 39, 45].

Husbands play various roles in facilitating or preventing their wives from accessing facility-based deliveries, ranging from: (a) persuading their wives to visit a facility and mobilizing the necessary transportation and funds [18, 25, 31, 39]; to (b) prohibiting a facility visit [17, 19, 24, 28]; to (c) playing a more neutral role [16, 19–22, 29, 39, 41, 47]. Husbands do not always hold the final authority – the husband’s decision-making power ranked below elder females across multiple contexts [17, 18, 21, 31].

Families with social connections to skilled providers may be more accepting of the biomedical approach to maternity care and thus more willing to seek a facility-based delivery. More importantly, a relative or friend working at a nearby facility can often arrange quicker admission or quality treatment of a parturient woman [20, 25, 32, 44].

### Ease of home birth

Home births are logistically easier than facility births and meet women’s desires to be surrounded by their belongings and the possibility of maintaining domestic responsibilities [18, 19, 21, 32, 33, 35, 42, 45, 46]. Although women may receive support in their domestic responsibilities from their neighbors [18], co-wives [19], or husbands [32], women were concerned that domestic chores would be neglected if they attended a health facility for delivery [18, 19, 21, 32, 33, 45].

### Effect of policies

Access to facility deliveries is influenced at a community or national level beyond the control of individual women. Seven studies addressed the effects of government policies and programs on a woman’s delivery location [21, 23, 31, 33, 34, 46, 48], including national health insurance schemes [23], social welfare programs [31, 33, 46, 48], population policies limiting the number of children allowed per couple [21], and national programs designed to increase facility-based deliveries [34].

### Resource availability and access

#### Transportation

Geographical distance and considerable travel times to health facilities are influential factors affecting women’s delivery locations [18, 20, 25–27, 29, 32–36, 39, 41, 45, 46, 49]. In contrast to the perceived inaccessibility of facilities, the accessibility of traditional practitioners may validate a woman’s decision to deliver at home. Likewise, limited availability of transportation options played a crucial role in whether or not a facility could be reached in a timely manner [25–27, 32, 36, 39, 41, 45, 47]. In the absence of a reliable private car or ambulance, women used arduous modes of transportation including bicycle, rickshaw, or public transportation. In some areas, local public transportation was the only means available, but services were often intermittent in rural areas and the cost of transportation was prohibitively expensive. Travel at night or on weekends is especially difficult as there are fewer options and higher costs [33, 39, 41, 43, 45, 46]. Furthermore, health facilities may be closed or lack appropriate staffing to manage a delivery or complications at night [33, 39]. Lack of access to transportation, good roads, adequate funds, and communication systems also make organizing referrals for obstetric complications a time-consuming process [17, 29, 34, 45, 49].

### Cost of childbirth

Direct costs associated with childbirth were prohibitively high for many women who viewed themselves as too poor to deliver in a facility [17, 20, 23, 24, 26, 28, 30, 32–37, 39, 41, 42, 46, 48, 49]. Low-resource households may have trouble acquiring funds to pay for facility-based care at the time-of-service, particularly those families who rely on seasonal labor [28, 32–34, 37, 41, 48, 49]. Collecting necessary funds were a difficult task as few moneylenders lent to the poor, and if they did, exorbitant interest rates could make the principle escalate rapidly [32, 37, 49]. Family members were often sent around the community to collect money from their neighbors [32, 37, 41, 48, 49].

Women viewed costs outside of the direct cost for a delivery as “hidden” and said they were difficult to prepare for [16, 19, 20, 24, 25, 30, 31, 34, 41, 43–45, 47–49]. Even in settings where direct delivery costs were subsidized, families were expected to pay for transportation to the facility, and other costs related to treatment at the facility [25, 30, 31, 34, 41, 43, 48, 49].

### Perceptions of quality of care

The perceived quality of care from providers affects a woman’s decisions on delivery location [16–18, 20, 21, 23, 24, 26, 28–31, 33–37, 39, 41, 42, 45–48]. “Perceived quality of care” differs from “quality of care” in that we captured the perspective of users and providers on the standard of care they experienced, as opposed to an independent assessment of the quality of care.

### Perceived quality of care from TBAs

Women emphasized the close bond they felt with TBAs, due to their status in the community and their trustworthiness [18, 23, 24, 30, 32, 33, 37, 39, 40, 43–46]. Some women believed that they received high quality care from TBAs and believed that TBAs played a supportive role [18, 19, 21, 22, 30, 33, 35, 40, 45]. However, women who believed TBAs provided low-quality care and did not trust their ability to handle complications were more inclined to seek facility-based care [20, 30, 33, 39, 43, 45]. Observing traditional practices did not preclude women from utilizing modern medical care [16–19, 22, 26, 32–34, 39, 44]. In medically pluralistic communities, many women moved freely between traditional and biomedical care models.

### Perceived quality of care at facilities

Some women viewed facilities as the safest and most respectable location for a delivery, believing that facilities were able to ensure positive outcomes [16–19, 21, 24, 29, 30, 33–35, 39, 41, 45, 47]. Furthermore, women who respected the competence of formal health workers and viewed them as “well-trained, competent, and compassionate” [19] “experts” [39] who provided “effective management of emergencies” [17] were likely to overcome various barriers to deliver in facilities.

However, women reporting negative interactions at facilities and lacking confidence in the health workers’ abilities, who they considered undertrained, incompetent, and inexperienced were less inclined to desire facility deliveries [17–19, 23, 26, 29–31, 36, 39, 43]. Women described providers as verbally and physically abusive, rude, bossy, disrespectful, insulting, easily angered, having poor attitudes, and lacking compassion [17–21, 24, 28, 34, 36, 37, 41, 42, 45–48]. Physical abuse included slapping, hitting, or forcefully holding women down. Negative interactions with providers were exacerbated for women of low socioeconomic status [20, 24, 28, 30, 48].

Women also experienced neglect and long delays in receiving facility-based care [17, 24, 31, 36, 39, 41, 45, 46, 48]. Health workers were slow to respond to patients’ needs and women reported feeling alone during delivery as health workers had poor communication skills and did not provide updates on labor progression [39].

Inadequate facility infrastructure and staffing contributed to an overall perception of low quality of care and many women complained of overcrowded wards without dedicated labor and delivery areas [17, 18, 24, 34, 35, 37, 39, 41, 45–47]. The lack of adequate staff also led to overburdened lower-level providers [17, 37, 39, 45, 47].

### Stigma

Women feared compulsory HIV-testing or HIV-testing without consent during facility-based delivery due to the fear of discrimination associated with a positive test [28, 30, 36, 38]. Some felt the only way to avoid HIV-testing was to deliver at home. The fear of unwanted HIV-status disclosure may prevent women from accessing facility delivery, as the lack of privacy in maternity wards impedes confidentiality [28, 36, 38]. Lastly, many communities view pregnancy and childbirth as the outcome of a marital relationship, thereby potentially stigmatizing and disempowering unwed women seeking facility delivery. Delivering at home was a desirable choice for unwed women or adolescents to avoid embarrassment or discrimination at a facility, particularly because these women were often lacking emotional and financial support from their partner or parents [17, 21, 41].

## Discussion

The emphasis placed by public health entities on increasing facility-based deliveries counters the commonly held belief among women and their families that childbirth is natural and need not be medicalized. Most communities studied in this review considered childbirth a natural event, and valued facilities primarily for the management of obstetric complications rather than as a default delivery location. When faced with the prospect of facility birth, women may fear various undesirable procedures, such as unfamiliar birthing positions, intrusive vaginal exams, and unnecessary surgical interventions. They may prefer home-based delivery with a TBA where they can maintain autonomy and supportive attendance.

The overarching themes presented in this review indicate that facilitators and barriers exist at multiple levels: within the woman’s control (perceptions of care), slightly outside of the woman’s control (family opinions and socioeconomic status), and at an institutional- and societal-level (policies and tradition). This paper synthesizes delivery experiences from 17 LMICs and identifies important similarities and differences in the decision-making process to seek facility-based care. Those familiar with obstetrics in LMICs may not find these findings surprising; however, the systematic and rigorous approach used in this review affords us more confidence in discussing higher-level themes across multiple contexts.

Twenty years ago, Thaddeus and Maine (1994) presented a framework identifying three phases of delay to accessing quality obstetric care: (a) delays in seeking care; (b) delays in reaching care; and (c) delays in receiving care [57]. Although the three-delays model is still valid, it may be too simplistic to explain why women still experience delays in accessing skilled delivery care. This review expands upon the three-delays model to illustrate how perceived quality of care by both traditional providers and facility-based providers influence the decision to seek care, as well as the impact of disrespect and abuse on delivery care-seeking behaviors. Public health programs to date have focused primarily on addressing resource availability and access issues to increase facility-based delivery rates. However, improving the quality of facility-based intrapartum care has the potential to further reduce the barriers to the utilization of facility-based delivery services.

Moving forward, we believe that future interventions should focus on achieving respectful, non-abusive, and high-quality intrapartum care for all women. This review highlighted several areas of disrespect and abuse by health workers. There has been a relative lack of research conducted on the definition, prevalence, and impact of disrespect and abuse during childbirth [58], and a further review is warranted to systematically synthesize existing evidence. Primary research should focus on identifying types of abuse and determining prevalence in different contexts. This will contribute to the development of operational definitions, validate measurement methods, and provide a gateway to develop evidence-based interventions to reduce disrespect and abuse during childbirth. Further research should be conducted to expand beyond the evaluation of intrapartum medical procedures to explore the effective implementation of such procedures in a humanized manner. Addressing concerns related to low-quality or disrespectful care at facilities would remove an important barrier to facility-birth for many women.

### Limitations of the review

We did not differentiate between types of “health facilities” in this review; rather we used the term as a proxy for skilled birth attendance because most included studies did not describe the facilities implicated in their research. Different levels of health facilities (i.e.: community health posts, district hospitals and referral centers) may have different facilitators and barriers associated with their use; however, it was not possible to disaggregate potential differences between types of facilities based on the included studies. Moreover, we did not include studies examining perspectives on having skilled birth attendants. Although skilled attendance during home birth is an alternative to facility-based birth in some contexts, we viewed it as conceptually different from facility-based deliveries with potentially different facilitators and barriers to use. Finally, this review presents a landscape of the factors influencing delivery choices, but not an evaluation of which factors are the most influential to an individual. Although no language filters were included in the search, six studies were excluded because they were not published in English or French.

## Conclusion

Accessing facility-based delivery care involves input from many actors and is influenced by myriad physical and sociocultural factors. Government policies, public health programs, and health workers encourage women to deliver in facilities, but women often yearn for the supportive attendance, privacy, and familiar practices that they experience while delivering at home. The desire for intergenerational continuity, the role of multiple actors in the decision-making process, and the perceived convenience of home births play crucial roles in the underutilization of facility-based care. Additionally, the inaccessibility of facilities due to geographical barriers and the high costs of facility-based delivery are critical barriers. Government policies, insurance schemes, and other public health programs often fail to effectively mitigate these physical barriers due to poor implementation. Finally, mistreatment, abuse, and neglect by health workers has fostered dissatisfaction, distrust, and avoidance of facility-based delivery care in many contexts.

Policy-makers and practitioners should work to strengthen the facilitators and mitigate the barriers described in order to increase facility-based deliveries in LMICs. This review highlights the need for improved, open communication between the healthcare system and the community. Some of the barriers that prevent women from attending the facility for childbirth could be addressed through providing purposive, direct information about the characteristics and potential benefits of facility-based delivery.

## Electronic supplementary material

Additional file 1: Appendices. (DOCX 128 KB)

Below are the links to the authors’ original submitted files for images.

Authors’ original file for figure 1

Authors’ original file for figure 2

Authors’ original file for figure 3

Authors’ original file for figure 4
