# Supplementary material for: Facilitators and barriers to facility-based delivery in low- and middle-income countries: a qualitative evidence synthesis
Source: Reprod Health. 2014 Sep 19;11:71. doi: 10.1186/1742-4755-11-71 (PMC4247708; doi:10.1186/1742-4755-11-71)
Supplement: Supplementary file 1 — Additional file 1: Appendices. (DOCX 128 KB) [file 12978_2014_330_MOESM1_ESM.docx]

**Appendix A - PubMed Search Strategy**

|  | **#** | **Searches** | **Results** |
| --- | --- | --- | --- |
| **MATERNAL / PERINATAL HEALTH** | 1 | “obstetric delivery”[tiab] OR "obstetric deliveries”[tiab] OR "delivery, obstetric"[Mesh] | 57582 |
|  | 2 | “perinatal care”[tiab] OR “peri natal care”[tiab] OR “perinatal healthcare”[tiab] OR “peri natal healthcare”[tiab] OR “perinatal health care”[tiab] OR “peri natal health care”[tiab] OR "perinatal care"[Mesh] | 7053 |
|  | 3 | “prenatal care”[tiab] OR "prenatal care"[Mesh] | 22225 |
|  | 4 | (“perinatal service”[tiab] OR “peri natal service”[tiab] OR “perinatal services”[tiab] OR “peri natal services”[tiab] OR “perinatal health service”[tiab] OR “peri natal health service”[tiab] OR “perinatal health services”[tiab] OR “peri natal health services”[tiab] OR “prenatal care”[tiab] OR “pre natal care”[tiab] OR “prenatal health care”[tiab] OR “prenatal healthcare”[tiab] OR “pre natal health care”[tiab] OR “pre natal healthcare”[tiab] OR “prenatal service”[tiab] OR “pre natal service”[tiab] OR “prenatal services”[tiab] OR “pre natal services”[tiab] OR “prenatal health service”[tiab] OR “pre natal health service”[tiab] OR “prenatal health services”[tiab] OR “pre natal health services”[tiab] OR “antenatal care”[tiab] OR “ante natal care”[tiab] OR “antenatal health care”[tiab] OR “antenatal healthcare”[tiab] OR “ante natal health care”[tiab] OR “ante natal healthcare”[tiab] OR “antenatal service”[tiab] OR “ante natal service”[tiab] OR “antenatal services”[tiab] OR “ante natal services”[tiab] OR “antenatal health service”[tiab] OR “ante natal health service”[tiab] OR “antenatal health services”[tiab] OR “ante natal health services”[tiab] OR “maternal care”[tiab] OR “maternal health care”[tiab] OR “maternal healthcare”[tiab] OR “maternal service”[tiab] OR “maternal health service”[tiab] OR “maternal services”[tiab] OR “maternal health services”[tiab]) AND (birth[tiab] OR births[tiab] OR childbirth[tiab] OR childbirths[tiab] OR delivery[tiab] OR deliveries[tiab]) | 6724 |
|  | 5 | prepare[tiab] OR prepares[tiab] OR preparing[tiab] OR preparation[tiab] OR preparations[tiab] | 382497 |
|  | 6 | birth[tiab] OR births[tiab] OR childbirth[tiab] OR “child birth”[tiab] OR childbirths[tiab] OR “child births”[tiab] | 208324 |
|  | 7 | #5 AND #6 | 2703 |
|  | 8 | #1 OR #2 OR #3 OR #4 OR #7 | 86591 |
| **FACILITIES** | 9 | "health facilities"[Mesh] | 561262 |
|  | 10 | “birthing centers"[tiab] OR "maternal-child health centers"[tiab] OR "delivery rooms"[tiab] OR "maternity hospitals"[tiab] | 1078 |
|  | 11 | #9 OR #10 | 561918 |
| **FACILITY-BASED DELIVERY** | 12 | “facility based delivery”[tiab] OR “facility based deliveries”[tiab] OR “facility delivery”[tiab] OR “facility deliveries”[tiab] OR “facility based births”[tiab] OR “facility based birth”[tiab] OR “facility birth”[tiab] OR “facility births”[tiab] OR “clinic delivery”[tiab] OR “clinic deliveries”[tiab] OR “clinic births”[tiab] OR “clinic birth”[tiab] OR “hospital delivery”[tiab] OR “hospital deliveries”[tiab] OR “hospital birth”[tiab] OR “hospital births”[tiab] OR “hospital childbirth”[tiab] OR “hospital childbirths”[tiab] OR “hospital based deliveries”[tiab] OR “hospital based delivery”[tiab] OR “hospital based births”[tiab] OR “institutional birth”[tiab] OR “institutional births”[tiab] OR “institutional childbirth”[tiab] OR “institutional childbirths”[tiab] OR “institutional delivery”[tiab] OR “institutional deliveries”[tiab] | 1239 |
|  | 13 | #8 AND #11 OR #12 | 6916 |
| **LMIC** | 14 | Developing Countries[Mesh:noexp] OR Africa[Mesh:noexp] OR Africa, Northern[Mesh:noexp] OR Africa South of the Sahara[Mesh:noexp] OR Africa, Central[Mesh:noexp] OR Africa, Eastern[Mesh:noexp] OR Africa, Southern[Mesh:noexp] OR Africa, Western[Mesh:noexp] OR Asia[Mesh:noexp] OR Asia, Central[Mesh:noexp] OR Asia, Southeastern[Mesh:noexp] OR Asia, Western[Mesh:noexp] OR Caribbean Region[Mesh:noexp] OR West Indies[Mesh:noexp] OR South America[Mesh:noexp] OR Latin America[Mesh:noexp] OR Central America[Mesh:noexp] OR Afghanistan[Mesh:noexp] OR Albania[Mesh:noexp] OR Algeria[Mesh:noexp] OR American Samoa[Mesh:noexp] OR Angola[Mesh:noexp] OR "Antigua and Barbuda"[Mesh:noexp] OR Argentina[Mesh:noexp] OR Armenia[Mesh:noexp] OR Azerbaijan[Mesh:noexp] OR Bahrain[Mesh:noexp] OR Bangladesh[Mesh:noexp] OR Barbados[Mesh:noexp] OR Benin[Mesh:noexp] OR Byelarus[Mesh:noexp] OR Belize[Mesh:noexp] OR Bhutan[Mesh:noexp] OR Bolivia[Mesh:noexp] OR Bosnia-Herzegovina[Mesh:noexp] OR Botswana[Mesh:noexp] OR Brazil[Mesh:noexp] OR Bulgaria[Mesh:noexp] OR Burkina Faso[Mesh:noexp] OR Burundi[Mesh:noexp] OR Cambodia[Mesh:noexp] OR Cameroon[Mesh:noexp] OR Cape Verde[Mesh:noexp] OR Central African Republic[Mesh:noexp] OR Chad[Mesh:noexp] OR Chile[Mesh:noexp] OR China[Mesh:noexp] OR Colombia[Mesh:noexp] OR Comoros[Mesh:noexp] OR Congo[Mesh:noexp] OR Costa Rica[Mesh:noexp] OR Cote d'Ivoire[Mesh:noexp] OR Croatia[Mesh:noexp] OR Cuba[Mesh:noexp] OR Cyprus[Mesh:noexp] OR Czechoslovakia[Mesh:noexp] OR Czech Republic[Mesh:noexp] OR Slovakia[Mesh:noexp] OR Djibouti[Mesh:noexp] OR "Democratic Republic of the Congo"[Mesh:noexp] OR Dominica[Mesh:noexp] OR Dominican Republic[Mesh:noexp] OR East Timor[Mesh:noexp] OR Ecuador[Mesh:noexp] OR Egypt[Mesh:noexp] OR El Salvador[Mesh:noexp] OR Eritrea[Mesh:noexp] OR Estonia[Mesh:noexp] OR Ethiopia[Mesh:noexp] OR Fiji[Mesh:noexp] OR Gabon[Mesh:noexp] OR Gambia[Mesh:noexp] OR "Georgia (Republic)"[Mesh:noexp] OR Ghana[Mesh:noexp] OR Greece[Mesh:noexp] OR Grenada[Mesh:noexp] OR Guatemala[Mesh:noexp] OR Guinea[Mesh:noexp] OR Guinea-Bissau[Mesh:noexp] OR Guam[Mesh:noexp] OR Guyana[Mesh:noexp] OR Haiti[Mesh:noexp] OR Honduras[Mesh:noexp] OR Hungary[Mesh:noexp] OR India[Mesh:noexp] OR Indonesia[Mesh:noexp] OR Iran[Mesh:noexp] OR Iraq[Mesh:noexp] OR Jamaica[Mesh:noexp] OR Jordan[Mesh:noexp] OR Kazakhstan[Mesh:noexp] OR Kenya[Mesh:noexp] OR Korea[Mesh:noexp] OR Kosovo[Mesh:noexp] OR Kyrgyzstan[Mesh:noexp] OR Laos[Mesh:noexp] OR Latvia[Mesh:noexp] OR Lebanon[Mesh:noexp] OR Lesotho[Mesh:noexp] OR Liberia[Mesh:noexp] OR Libya[Mesh:noexp] OR Lithuania[Mesh:noexp] OR Macedonia[Mesh:noexp] OR Madagascar[Mesh:noexp] OR Malaysia[Mesh:noexp] OR Malawi[Mesh:noexp] OR Mali[Mesh:noexp] OR Malta[Mesh:noexp] OR Mauritania[Mesh:noexp] OR Mauritius[Mesh:noexp] OR Mexico[Mesh:noexp] OR Micronesia[Mesh:noexp] OR Middle East[Mesh:noexp] OR Moldova[Mesh:noexp] OR Mongolia[Mesh:noexp] OR Montenegro[Mesh:noexp] OR Morocco[Mesh:noexp] OR Mozambique[Mesh:noexp] OR Myanmar[Mesh:noexp] OR Namibia[Mesh:noexp] OR Nepal[Mesh:noexp] OR Netherlands Antilles[Mesh:noexp] OR New Caledonia[Mesh:noexp] OR Nicaragua[Mesh:noexp] OR Niger[Mesh:noexp] OR Nigeria[Mesh:noexp] OR Oman[Mesh:noexp] OR Pakistan[Mesh:noexp] OR Palau[Mesh:noexp] OR Panama[Mesh:noexp] OR Papua New Guinea[Mesh:noexp] OR Paraguay[Mesh:noexp] OR Peru[Mesh:noexp] OR Philippines[Mesh:noexp] OR Poland[Mesh:noexp] OR Portugal[Mesh:noexp] OR Puerto Rico[Mesh:noexp] OR Romania[Mesh:noexp] OR Russia[Mesh:noexp] OR "Russia (Pre-1917)"[Mesh:noexp] OR Rwanda[Mesh:noexp] OR "Saint Kitts and Nevis"[Mesh:noexp] OR Saint Lucia[Mesh:noexp] OR "Saint Vincent and the Grenadines"[Mesh:noexp] OR Samoa[Mesh:noexp] OR Saudi Arabia[Mesh:noexp] OR Senegal[Mesh:noexp] OR Serbia[Mesh:noexp] OR Montenegro[Mesh:noexp] OR Seychelles[Mesh:noexp] OR Sierra Leone[Mesh:noexp] OR Slovenia[Mesh:noexp] OR Sri Lanka[Mesh:noexp] OR Somalia[Mesh:noexp] OR South Africa[Mesh:noexp] OR Sudan[Mesh:noexp] OR Suriname[Mesh:noexp] OR Swaziland[Mesh:noexp] OR Syria[Mesh:noexp] OR Tajikistan[Mesh:noexp] OR Tanzania[Mesh:noexp] OR Thailand[Mesh:noexp] OR Togo[Mesh:noexp] OR Tonga[Mesh:noexp] OR "Trinidad and Tobago"[Mesh:noexp] OR Tunisia[Mesh:noexp] OR Turkey[Mesh:noexp] OR Turkmenistan[Mesh:noexp] OR Uganda[Mesh:noexp] OR Ukraine[Mesh:noexp] OR Uruguay[Mesh:noexp] OR USSR[Mesh:noexp] OR Uzbekistan[Mesh:noexp] OR Vanuatu[Mesh:noexp] OR Venezuela[Mesh:noexp] OR Vietnam[Mesh:noexp] OR Yemen[Mesh:noexp] OR Yugoslavia[Mesh:noexp] OR Zambia[Mesh:noexp] OR Zimbabwe[Mesh:noexp] | 823291 |
|  | 15 | Macedonia[ot] OR Madagascar[ot] OR Malagasy Republic[ot] OR Malaysia[ot] OR Malaya[ot] OR Malay[ot] OR Sabah[ot] OR Sarawak[ot] OR Malawi[ot] OR Nyasaland[ot] OR Mali[ot] OR Malta[ot] OR Marshall Islands[ot] OR Mauritania[ot] OR Mauritius[ot] OR Agalega Islands[ot] OR Mexico[ot] OR Micronesia[ot] OR Middle East[ot] OR Moldova[ot] OR Moldovia[ot] OR Moldovian[ot] OR Mongolia[ot] OR Montenegro[ot] OR Morocco[ot] OR Ifni[ot] OR Mozambique[ot] OR Myanmar[ot] OR Myanma[ot] OR Burma[ot] OR Namibia[ot] OR Nepal[ot] OR Netherlands Antilles[ot] OR New Caledonia[ot] OR Nicaragua[ot] OR Niger[ot] OR Nigeria[ot] OR Northern Mariana Islands[ot] OR Oman[ot] OR Muscat[ot] OR Pakistan[ot] OR Palau[ot] OR Palestine[ot] OR Panama[ot] OR Paraguay[ot] OR Peru[ot] OR Philippines[ot] OR Philipines[ot] OR Phillipines[ot] OR Phillippines[ot] OR Poland[ot] OR Portugal[ot] OR Puerto Rico[ot] OR Romania[ot] OR Rumania[ot] OR Roumania[ot] OR Russia[ot] OR Russian[ot] OR Rwanda[ot] OR Ruanda[ot] OR Saint Kitts[ot] OR St Kitts[ot] OR Nevis[ot] OR Saint Lucia[ot] OR St Lucia[ot] OR Saint Vincent[ot] OR St Vincent[ot] OR Grenadines[ot] OR Samoa[ot] OR Samoan Islands[ot] OR Navigator Island[ot] OR Navigator Islands[ot] OR Sao Tome[ot] OR Saudi Arabia[ot] OR Senegal[ot] OR Serbia[ot] OR Montenegro[ot] OR Seychelles[ot] OR Sierra Leone[ot] OR Slovenia[ot] OR Sri Lanka[ot] OR Ceylon[ot] OR Solomon Islands[ot] OR Somalia[ot] OR Sudan[ot] OR Suriname[ot] OR Surinam[ot] OR Swaziland[ot] OR Syria[ot] OR Tajikistan[ot] OR Tadzhikistan[ot] OR Tadjikistan[ot] OR Tadzhik[ot] OR Tanzania[ot] OR Thailand[ot] OR Togo[ot] OR Togolese Republic[ot] OR Tonga[ot] OR Trinidad[ot] OR Tobago[ot] OR Tunisia[ot] OR Turkey[ot] OR Turkmenistan[ot] OR Turkmen[ot] OR Uganda[ot] OR Ukraine[ot] OR Uruguay[ot] OR USSR[ot] OR Soviet Union[ot] OR Union of Soviet Socialist Republics[ot] OR Uzbekistan[ot] OR Uzbek OR Vanuatu[ot] OR New Hebrides[ot] OR Venezuela[ot] OR Vietnam[ot] OR Viet Nam[ot] OR West Bank[ot] OR Yemen[ot] OR Yugoslavia[ot] OR Zambia[ot] OR Zimbabwe[ot] OR Rhodesia[ot] | 18502 |
|  | 16 | Africa[ot] OR Asia[ot] OR Caribbean[ot] OR West Indies[ot] OR South America[ot] OR Latin America[ot] OR Central America[ot] OR Afghanistan[ot] OR Albania[ot] OR Algeria[ot] OR Angola[ot] OR Antigua[ot] OR Barbuda[ot] OR Argentina[ot] OR Armenia[ot] OR Armenian[ot] OR Aruba[ot] OR Azerbaijan[ot] OR Bahrain[ot] OR Bangladesh[ot] OR Barbados[ot] OR Benin[ot] OR Byelarus[ot] OR Byelorussian[ot] OR Belarus[ot] OR Belorussian[ot] OR Belorussia[ot] OR Belize[ot] OR Bhutan[ot] OR Bolivia[ot] OR Bosnia[ot] OR Herzegovina[ot] OR Hercegovina[ot] OR Botswana[ot] OR Brasil[ot] OR Brazil[ot] OR Bulgaria[ot] OR Burkina Faso[ot] OR Burkina Fasso[ot] OR Upper Volta[ot] OR Burundi[ot] OR Urundi[ot] OR Cambodia[ot] OR Khmer Republic[ot] OR Kampuchea[ot] OR Cameroon[ot] OR Cameroons[ot] OR Cameron[ot] OR Camerons[ot] OR Cape Verde[ot] OR Central African Republic[ot] OR Chad[ot] OR Chile[ot] OR China[ot] OR Colombia[ot] OR Comoros[ot] OR Comoro Islands[ot] OR Comores[ot] OR Mayotte[ot] OR Congo[ot] OR Zaire[ot] OR Costa Rica[ot] OR Cote d'Ivoire[ot] OR Ivory Coast[ot] OR Croatia[ot] OR Cuba[ot] OR Cyprus[ot] OR Czechoslovakia[ot] OR Czech Republic[ot] OR Slovakia[ot] OR Slovak Republic[ot] OR Djibouti[ot] OR French Somaliland[ot] OR Dominica[ot] OR Dominican Republic[ot] OR East Timor[ot] OR East Timur[ot] OR Timor Leste[ot] OR Ecuador[ot] OR Egypt[ot] OR United Arab Republic[ot] OR El Salvador[ot] OR Eritrea[ot] OR Estonia[ot] OR Ethiopia[ot] OR Fiji[ot] OR Gabon[ot] OR Gabonese Republic[ot] OR Gambia[ot] OR Gaza[ot] OR "Georgia Republic"[ot] OR "Georgian Republic"[ot] OR Ghana[ot] OR Gold Coast[ot] OR Greece[ot] OR Grenada[ot] OR Guatemala[ot] OR Guinea[ot] OR Guam[ot] OR Guiana[ot] OR Guyana[ot] OR Haiti[ot] OR Honduras[ot] OR Hungary[ot] OR India[ot] OR Maldives[ot] OR Indonesia[ot] OR Iran[ot] OR Iraq[ot] OR Isle of Man[ot] OR Jamaica[ot] OR Jordan[ot] OR Kazakhstan[ot] OR Kazakh[ot] OR Kenya[ot] OR Kiribati[ot] OR Korea[ot] OR Kosovo[ot] OR Kyrgyzstan[ot] OR Kirghizia[ot] OR Kyrgyz Republic[ot] OR Kirghiz[ot] OR Kirgizstan[ot] OR "Lao PDR"[ot] OR Laos[ot] OR Latvia[ot] OR Lebanon[ot] OR Lesotho[ot] OR Basutoland[ot] OR Liberia[ot] OR Libya[ot] OR Lithuania[ot] | 41163 |
|  | 17 | Macedonia[tiab] OR Madagascar[tiab] OR Malagasy Republic[tiab] OR Malaysia[tiab] OR Malaya[tiab] OR Malay[tiab] OR Sabah[tiab] OR Sarawak[tiab] OR Malawi[tiab] OR Nyasaland[tiab] OR Mali[tiab] OR Malta[tiab] OR Marshall Islands[tiab] OR Mauritania[tiab] OR Mauritius[tiab] OR Agalega Islands[tiab] OR Mexico[tiab] OR Micronesia[tiab] OR Middle East[tiab] OR Moldova[tiab] OR Moldovia[tiab] OR Moldovian[tiab] OR Mongolia[tiab] OR Montenegro[tiab] OR Morocco[tiab] OR Ifni[tiab] OR Mozambique[tiab] OR Myanmar[tiab] OR Myanma[tiab] OR Burma[tiab] OR Namibia[tiab] OR Nepal[tiab] OR Netherlands Antilles[tiab] OR New Caledonia[tiab] OR Nicaragua[tiab] OR Niger[tiab] OR Nigeria[tiab] OR Northern Mariana Islands[tiab] OR Oman[tiab] OR Muscat[tiab] OR Pakistan[tiab] OR Palau[tiab] OR Palestine[tiab] OR Panama[tiab] OR Paraguay[tiab] OR Peru[tiab] OR Philippines[tiab] OR Philipines[tiab] OR Phillipines[tiab] OR Phillippines[tiab] OR Poland[tiab] OR Portugal[tiab] OR Puerto Rico[tiab] OR Romania[tiab] OR Rumania[tiab] OR Roumania[tiab] OR Russia[tiab] OR Russian[tiab] OR Rwanda[tiab] OR Ruanda[tiab] OR Saint Kitts[tiab] OR St Kitts[tiab] OR Nevis[tiab] OR Saint Lucia[tiab] OR St Lucia[tiab] OR Saint Vincent[tiab] OR St Vincent[tiab] OR Grenadines[tiab] OR Samoa[tiab] OR Samoan Islands[tiab] OR Navigator Island[tiab] OR Navigator Islands[tiab] OR Sao Tome[tiab] OR Saudi Arabia[tiab] OR Senegal[tiab] OR Serbia[tiab] OR Montenegro[tiab] OR Seychelles[tiab] OR Sierra Leone[tiab] OR Slovenia[tiab] OR Sri Lanka[tiab] OR Ceylon[tiab] OR Solomon Islands[tiab] OR Somalia[tiab] OR Sudan[tiab] OR Suriname[tiab] OR Surinam[tiab] OR Swaziland[tiab] OR Syria[tiab] OR Tajikistan[tiab] OR Tadzhikistan[tiab] OR Tadjikistan[tiab] OR Tadzhik[tiab] OR Tanzania[tiab] OR Thailand[tiab] OR Togo[tiab] OR Togolese Republic[tiab] OR Tonga[tiab] OR Trinidad[tiab] OR Tobago[tiab] OR Tunisia[tiab] OR Turkey[tiab] OR Turkmenistan[tiab] OR Turkmen[tiab] OR Uganda[tiab] OR Ukraine[tiab] OR Uruguay[tiab] OR USSR[tiab] OR Soviet Union[tiab] OR Union of Soviet Socialist Republics[tiab] OR Uzbekistan[tiab] OR Uzbek OR Vanuatu[tiab] OR New Hebrides[tiab] OR Venezuela[tiab] OR Vietnam[tiab] OR Viet Nam[tiab] OR West Bank[tiab] OR Yemen[tiab] OR Yugoslavia[tiab] OR Zambia[tiab] OR Zimbabwe[tiab] OR Rhodesia[tiab] | 253625 |
|  | 18 | Africa[tiab] OR Asia[tiab] OR Caribbean[tiab] OR West Indies[tiab] OR South America[tiab] OR Latin America[tiab] OR Central America[tiab] OR Afghanistan[tiab] OR Albania[tiab] OR Algeria[tiab] OR Angola[tiab] OR Antigua[tiab] OR Barbuda[tiab] OR Argentina[tiab] OR Armenia[tiab] OR Armenian[tiab] OR Aruba[tiab] OR Azerbaijan[tiab] OR Bahrain[tiab] OR Bangladesh[tiab] OR Barbados[tiab] OR Benin[tiab] OR Byelarus[tiab] OR Byelorussian[tiab] OR Belarus[tiab] OR Belorussian[tiab] OR Belorussia[tiab] OR Belize[tiab] OR Bhutan[tiab] OR Bolivia[tiab] OR Bosnia[tiab] OR Herzegovina[tiab] OR Hercegovina[tiab] OR Botswana[tiab] OR Brasil[tiab] OR Brazil[tiab] OR Bulgaria[tiab] OR Burkina Faso[tiab] OR Burkina Fasso[tiab] OR Upper Volta[tiab] OR Burundi[tiab] OR Urundi[tiab] OR Cambodia[tiab] OR Khmer Republic[tiab] OR Kampuchea[tiab] OR Cameroon[tiab] OR Cameroons[tiab] OR Cameron[tiab] OR Camerons[tiab] OR Cape Verde[tiab] OR Central African Republic[tiab] OR Chad[tiab] OR Chile[tiab] OR China[tiab] OR Colombia[tiab] OR Comoros[tiab] OR Comoro Islands[tiab] OR Comores[tiab] OR Mayotte[tiab] OR Congo[tiab] OR Zaire[tiab] OR Costa Rica[tiab] OR Cote d'Ivoire[tiab] OR Ivory Coast[tiab] OR Croatia[tiab] OR Cuba[tiab] OR Cyprus[tiab] OR Czechoslovakia[tiab] OR Czech Republic[tiab] OR Slovakia[tiab] OR Slovak Republic[tiab] OR Djibouti[tiab] OR French Somaliland[tiab] OR Dominica[tiab] OR Dominican Republic[tiab] OR East Timor[tiab] OR East Timur[tiab] OR Timor Leste[tiab] OR Ecuador[tiab] OR Egypt[tiab] OR United Arab Republic[tiab] OR El Salvador[tiab] OR Eritrea[tiab] OR Estonia[tiab] OR Ethiopia[tiab] OR Fiji[tiab] OR Gabon[tiab] OR Gabonese Republic[tiab] OR Gambia[tiab] OR Gaza[tiab] OR Georgia Republic[tiab] OR Georgian Republic[tiab] OR Ghana[tiab] OR Gold Coast[tiab] OR Greece[tiab] OR Grenada[tiab] OR Guatemala[tiab] OR Guinea[tiab] OR Guam[tiab] OR Guiana[tiab] OR Guyana[tiab] OR Haiti[tiab] OR Honduras[tiab] OR Hungary[tiab] OR India[tiab] OR Maldives[tiab] OR Indonesia[tiab] OR Iran[tiab] OR Iraq[tiab] OR Isle of Man[tiab] OR Jamaica[tiab] OR Jordan[tiab] OR Kazakhstan[tiab] OR Kazakh[tiab] OR Kenya[tiab] OR Kiribati[tiab] OR Korea[tiab] OR Kosovo[tiab] OR Kyrgyzstan[tiab] OR Kirghizia[tiab] OR Kyrgyz Republic[tiab] OR Kirghiz[tiab] OR Kirgizstan[tiab] OR "Lao PDR"[tiab] OR Laos[tiab] OR Latvia[tiab] OR Lebanon[tiab] OR Lesotho[tiab] OR Basutoland[tiab] OR Liberia[tiab] OR Libya[tiab] OR Lithuania[tiab] | 486944 |
|  | 19 | Macedonia[pl] OR Madagascar[pl] OR Malagasy Republic[pl] OR Malaysia[pl] OR Malaya[pl] OR Malay[pl] OR Sabah[pl] OR Sarawak[pl] OR Malawi[pl] OR Nyasaland[pl] OR Mali[pl] OR Malta[pl] OR Marshall Islands[pl] OR Mauritania[pl] OR Mauritius[pl] OR Agalega Islands[pl] OR Mexico[pl] OR Micronesia[pl] OR Middle East[pl] OR Moldova[pl] OR Moldovia[pl] OR Moldovian[pl] OR Mongolia[pl] OR Montenegro[pl] OR Morocco[pl] OR Ifni[pl] OR Mozambique[pl] OR Myanmar[pl] OR Myanma[pl] OR Burma[pl] OR Namibia[pl] OR Nepal[pl] OR Netherlands Antilles[pl] OR New Caledonia[pl] OR Nicaragua[pl] OR Niger[pl] OR Nigeria[pl] OR Northern Mariana Islands[pl] OR Oman[pl] OR Muscat[pl] OR Pakistan[pl] OR Palau[pl] OR Palestine[pl] OR Panama[pl] OR Paraguay[pl] OR Peru[pl] OR Philippines[pl] OR Philipines[pl] OR Phillipines[pl] OR Phillippines[pl] OR Poland[pl] OR Portugal[pl] OR Puerto Rico[pl] OR Romania[pl] OR Rumania[pl] OR Roumania[pl] OR Russia[pl] OR Russian[pl] OR Rwanda[pl] OR Ruanda[pl] OR Saint Kitts[pl] OR St Kitts[pl] OR Nevis[pl] OR Saint Lucia[pl] OR St Lucia[pl] OR Saint Vincent[pl] OR St Vincent[pl] OR Grenadines[pl] OR Samoa[pl] OR Samoan Islands[pl] OR Navigator Island[pl] OR Navigator Islands[pl] OR Sao Tome[pl] OR Saudi Arabia[pl] OR Senegal[pl] OR Serbia[pl] OR Montenegro[pl] OR Seychelles[pl] OR Sierra Leone[pl] OR Slovenia[pl] OR Sri Lanka[pl] OR Ceylon[pl] OR Solomon Islands[pl] OR Somalia[pl] OR South Africa[pl] OR Sudan[pl] OR Suriname[pl] OR Surinam[pl] OR Swaziland[pl] OR Syria[pl] OR Tajikistan[pl] OR Tadzhikistan[pl] OR Tadjikistan[pl] OR Tadzhik[pl] OR Tanzania[pl] OR Thailand[pl] OR Togo[pl] OR Togolese Republic[pl] OR Tonga[pl] OR Trinidad[pl] OR Tobago[pl] OR Tunisia[pl] OR Turkey[pl] OR Turkmenistan[pl] OR Turkmen[pl] OR Uganda[pl] OR Ukraine[pl] OR Uruguay[pl] OR USSR[pl] OR Soviet Union[pl] OR Union of Soviet Socialist Republics[pl] OR Uzbekistan[pl] OR Uzbek OR Vanuatu[pl] OR New Hebrides[pl] OR Venezuela[pl] OR Vietnam[pl] OR Viet Nam[pl] OR West Bank[pl] OR Yemen[pl] OR Yugoslavia[pl] OR Zambia[pl] OR Zimbabwe[pl] OR Rhodesia[pl] | 1091395 |
|  | 20 | Africa[pl] OR Asia[pl] OR Caribbean[pl] OR West Indies[pl] OR South America[pl] OR Latin America[pl] OR Central America[pl] OR Afghanistan[pl] OR Albania[pl] OR Algeria[pl] OR Angola[pl] OR Antigua[pl] OR Barbuda[pl] OR Argentina[pl] OR Armenia[pl] OR Armenian[pl] OR Aruba[pl] OR Azerbaijan[pl] OR Bahrain[pl] OR Bangladesh[pl] OR Barbados[pl] OR Benin[pl] OR Byelarus[pl] OR Byelorussian[pl] OR Belarus[pl] OR Belorussian[pl] OR Belorussia[pl] OR Belize[pl] OR Bhutan[pl] OR Bolivia[pl] OR Bosnia[pl] OR Herzegovina[pl] OR Hercegovina[pl] OR Botswana[pl] OR Brasil[pl] OR Brazil[pl] OR Bulgaria[pl] OR Burkina Faso[pl] OR Burkina Fasso[pl] OR Upper Volta[pl] OR Burundi[pl] OR Urundi[pl] OR Cambodia[pl] OR Khmer Republic[pl] OR Kampuchea[pl] OR Cameroon[pl] OR Cameroons[pl] OR Cameron[pl] OR Camerons[pl] OR Cape Verde[pl] OR Central African Republic[pl] OR Chad[pl] OR Chile[pl] OR China[pl] OR Colombia[pl] OR Comoros[pl] OR Comoro Islands[pl] OR Comores[pl] OR Mayotte[pl] OR Congo[pl] OR Zaire[pl] OR Costa Rica[pl] OR Cote d'Ivoire[pl] OR Ivory Coast[pl] OR Croatia[pl] OR Cuba[pl] OR Cyprus[pl] OR Czechoslovakia[pl] OR Czech Republic[pl] OR Slovakia[pl] OR Slovak Republic[pl] OR Djibouti[pl] OR French Somaliland[pl] OR Dominica[pl] OR Dominican Republic[pl] OR East Timor[pl] OR East Timur[pl] OR Timor Leste[pl] OR Ecuador[pl] OR Egypt[pl] OR United Arab Republic[pl] OR El Salvador[pl] OR Eritrea[pl] OR Estonia[pl] OR Ethiopia[pl] OR Fiji[pl] OR Gabon[pl] OR Gabonese Republic[pl] OR Gambia[pl] OR Gaza[pl] OR Georgia Republic[pl] OR Georgian Republic[pl] OR Ghana[pl] OR Gold Coast[pl] OR Greece[pl] OR Grenada[pl] OR Guatemala[pl] OR Guinea[pl] OR Guam[pl] OR Guiana[pl] OR Guyana[pl] OR Haiti[pl] OR Honduras[pl] OR Hungary[pl] OR India[pl] OR Maldives[pl] OR Indonesia[pl] OR Iran[pl] OR Iraq[pl] OR Isle of Man[pl] OR Jamaica[pl] OR Jordan[pl] OR Kazakhstan[pl] OR Kazakh[pl] OR Kenya[pl] OR Kiribati[pl] OR Korea[pl] OR Kosovo[pl] OR Kyrgyzstan[pl] OR Kirghizia[pl] OR Kyrgyz Republic[pl] OR Kirghiz[pl] OR Kirgizstan[pl] OR "Lao PDR"[pl] OR Laos[pl] OR Latvia[pl] OR Lebanon[pl] OR Lesotho[pl] OR Basutoland[pl] OR Liberia[pl] OR Libya[pl] OR Lithuania[pl] | 975498 |
|  | 21 | "developing country"[ot] OR "developing countries"[ot] OR "developing nation"[ot] OR "developing nations"[ot] OR "developing population"[ot] OR "developing populations"[ot] OR "developing world"[ot] OR "less developed country"[ot] OR "less developed countries"[ot] OR "less developed nation"[ot] OR "less developed nations"[ot] OR "less developed population"[ot] OR "less developed populations"[ot] OR "less developed world"[ot] OR "lesser developed country"[ot] OR "lesser developed countries"[ot] OR "lesser developed nation"[ot] OR "lesser developed nations"[ot] OR "lesser developed population"[ot] OR "lesser developed populations"[ot] OR "lesser developed world"[ot] OR "under developed country"[ot] OR "under developed countries"[ot] OR "under developed nation"[ot] OR "under developed nations"[ot] OR "under developed population"[ot] OR "under developed populations"[ot] OR "under developed world"[ot] OR "underdeveloped country"[ot] OR "underdeveloped countries"[ot] OR "underdeveloped nation"[ot] OR "underdeveloped nations"[ot] OR "underdeveloped population"[ot] OR "underdeveloped populations"[ot] OR "underdeveloped world"[ot] OR "middle income country"[ot] OR "middle income countries"[ot] OR "middle income nation"[ot] OR "middle income nations"[ot] OR "middle income population"[ot] OR "middle income populations"[ot] OR "low income country"[ot] OR "low income countries"[ot] OR "low income nation"[ot] OR "low income nations"[ot] OR "low income population"[ot] OR "low income populations"[ot] OR "lower income country"[ot] OR "lower income countries"[ot] OR "lower income nation"[ot] OR "lower income nations"[ot] OR "lower income population"[ot] OR "lower income populations"[ot] OR "underserved country"[ot] OR "underserved countries"[ot] OR "underserved nation"[ot] OR "underserved nations"[ot] OR "underserved population"[ot] OR "underserved populations"[ot] OR "underserved world"[ot] OR "under served country"[ot] OR "under served countries"[ot] OR "under served nation"[ot] OR "under served nations"[ot] OR "under served population"[ot] OR "under served populations"[ot] OR "under served world"[ot] OR "deprived country"[ot] OR "deprived countries"[ot] OR "deprived nation"[ot] OR "deprived nations"[ot] OR "deprived population"[ot] OR "deprived populations"[ot] OR "deprived world"[ot] OR "poor country"[ot] OR "poor countries"[ot] OR "poor nation"[ot] OR "poor nations"[ot] OR "poor population"[ot] OR "poor populations"[ot] OR "poor world"[ot] OR "poorer country"[ot] OR "poorer countries"[ot] OR "poorer nation"[ot] OR "poorer nations"[ot] OR "poorer population"[ot] OR "poorer populations"[ot] OR "poorer world"[ot] OR "developing economy"[ot] OR "developing economies"[ot] OR "less developed economy"[ot] OR "less developed economies"[ot] OR "lesser developed economy"[ot] OR "lesser developed economies"[ot] OR "under developed economy"[ot] OR "under developed economies"[ot] OR "underdeveloped economy"[ot] OR "underdeveloped economies"[ot] OR "middle income economy"[ot] OR "middle income economies"[ot] OR "low income economy"[ot] OR "low income economies"[ot] OR "lower income economy"[ot] OR "lower income economies"[ot] OR "low gdp"[ot] OR "low gnp"[ot] OR "low gross domestic"[ot] OR "low gross national"[ot] OR "lower gdp"[ot] OR "lower gnp"[ot] OR "lower gross domestic"[ot] OR "lower gross national"[ot] OR lmic[ot] OR lmics[ot] OR "third world"[ot] OR "lami country"[ot] OR "lami countries"[ot] OR "transitional country"[ot] OR "transitional countries"[ot] | 31224 |
|  | 22 | "developing country"[tiab] OR "developing countries"[tiab] OR "developing nation"[tiab] OR "developing nations"[tiab] OR "developing population"[tiab] OR "developing populations"[tiab] OR "developing world"[tiab] OR "less developed country"[tiab] OR "less developed countries"[tiab] OR "less developed nation"[tiab] OR "less developed nations"[tiab] OR "less developed population"[tiab] OR "less developed populations"[tiab] OR "less developed world"[tiab] OR "lesser developed country"[tiab] OR "lesser developed countries"[tiab] OR "lesser developed nation"[tiab] OR "lesser developed nations"[tiab] OR "lesser developed population"[tiab] OR "lesser developed populations"[tiab] OR "lesser developed world"[tiab] OR "under developed country"[tiab] OR "under developed countries"[tiab] OR "under developed nation"[tiab] OR "under developed nations"[tiab] OR "under developed population"[tiab] OR "under developed populations"[tiab] OR "under developed world"[tiab] OR "underdeveloped country"[tiab] OR "underdeveloped countries"[tiab] OR "underdeveloped nation"[tiab] OR "underdeveloped nations"[tiab] OR "underdeveloped population"[tiab] OR "underdeveloped populations"[tiab] OR "underdeveloped world"[tiab] OR "middle income country"[tiab] OR "middle income countries"[tiab] OR "middle income nation"[tiab] OR "middle income nations"[tiab] OR "middle income population"[tiab] OR "middle income populations"[tiab] OR "low income country"[tiab] OR "low income countries"[tiab] OR "low income nation"[tiab] OR "low income nations"[tiab] OR "low income population"[tiab] OR "low income populations"[tiab] OR "lower income country"[tiab] OR "lower income countries"[tiab] OR "lower income nation"[tiab] OR "lower income nations"[tiab] OR "lower income population"[tiab] OR "lower income populations"[tiab] OR "underserved country"[tiab] OR "underserved countries"[tiab] OR "underserved nation"[tiab] OR "underserved nations"[tiab] OR "underserved population"[tiab] OR "underserved populations"[tiab] OR "underserved world"[tiab] OR "under served country"[tiab] OR "under served countries"[tiab] OR "under served nation"[tiab] OR "under served nations"[tiab] OR "under served population"[tiab] OR "under served populations"[tiab] OR "under served world"[tiab] OR "deprived country"[tiab] OR "deprived countries"[tiab] OR "deprived nation"[tiab] OR "deprived nations"[tiab] OR "deprived population"[tiab] OR "deprived populations"[tiab] OR "deprived world"[tiab] OR "poor country"[tiab] OR "poor countries"[tiab] OR "poor nation"[tiab] OR "poor nations"[tiab] OR "poor population"[tiab] OR "poor populations"[tiab] OR "poor world"[tiab] OR "poorer country"[tiab] OR "poorer countries"[tiab] OR "poorer nation"[tiab] OR "poorer nations"[tiab] OR "poorer population"[tiab] OR "poorer populations"[tiab] OR "poorer world"[tiab] OR "developing economy"[tiab] OR "developing economies"[tiab] OR "less developed economy"[tiab] OR "less developed economies"[tiab] OR "lesser developed economy"[tiab] OR "lesser developed economies"[tiab] OR "under developed economy"[tiab] OR "under developed economies"[tiab] OR "underdeveloped economy"[tiab] OR "underdeveloped economies"[tiab] OR "middle income economy"[tiab] OR "middle income economies"[tiab] OR "low income economy"[tiab] OR "low income economies"[tiab] OR "lower income economy"[tiab] OR "lower income economies"[tiab] OR "low gdp"[tiab] OR "low gnp"[tiab] OR "low gross domestic"[tiab] OR "low gross national"[tiab] OR "lower gdp"[tiab] OR "lower gnp"[tiab] OR "lower gross domestic"[tiab] OR "lower gross national"[tiab] OR lmic[tiab] OR lmics[tiab] OR "third world"[tiab] OR "lami country"[tiab] OR "lami countries"[tiab] OR "transitional country"[tiab] OR "transitional countries"[tiab] | 52275 |
|  | 23 | #14 OR #15 OR #16 OR #17 OR #18 OR #19 OR #20 OR #21 OR #22 | 2788719 |
| **QUALITATIVE RESEARCH** | 24 | “qualitative research”[mesh] OR “qualitative research”[tiab] OR “qualitative study”[tiab] OR “qualitative studies”[tiab] OR “focus groups”[mesh] OR “focus group”[tiab] OR “focus groups”[tiab] OR “interviews as topic”[mesh] OR interview[tiab] OR “interviews”[tiab] OR “health services administration”[mh] OR “group discussion”[tiab] OR “group discussions”[tiab] | 2079139 |
|  | 25 | ("semi-structured"[tiab] OR semistructured[tiab] OR unstructured[tiab] OR informal[tiab] OR "in-depth"[tiab] OR indepth[tiab] OR "face-to-face"[tiab] OR structured[tiab] OR guide[tiab] OR guides[tiab]) AND (interview*[tiab] OR discussion*[tiab] OR questionnaire*[tiab]) | 70062 |
|  | 26 | “focus group*”[tiab] OR qualitative[tiab] OR ethnograph*[tiab] OR fieldwork[tiab] OR "field work"[tiab] OR "key informant"[tiab] | 119354 |
|  | 27 | #24 OR #25 OR #26 | 2156792 |
|  | 28 | #13 AND #23 AND #27 | 1408 |

**Appendix B - CINAHL Search Strategy**

| # | Searches | Results |
| --- | --- | --- |
| 1 | (MH "Maternal-Child Care+") OR (MH “Obstetric Patients+”) OR (MH “Rooming In+”) OR (MH “Obstetric Service+”) OR (MH “Childbirth+”) OR TI “perinatal care” OR TI “perinatal service” OR TI “perinatal services” OR TI “peri natal care” OR TI “peri natal service” OR TI “peri natal services” OR TI “prenatal care” OR TI “prenatal service” OR TI “prenatal services” OR TI “pre natal care” OR TI “pre natal service” OR TI “pre natal services” OR TI “antenatal care” OR TI “antenatal service” OR TI “antenatal services” OR TI “ante natal care” OR TI “ante natal service” OR TI “ante natal services” OR TI “maternal care” OR TI “maternal service” OR TI “maternal services” OR TI childbirth OR TI childbirths OR AB “perinatal care” OR AB “perinatal service” OR AB “perinatal services” OR AB “peri natal care” OR AB “peri natal service” OR AB “peri natal services” OR AB “prenatal care” OR AB “prenatal service” OR AB “prenatal services” OR AB “pre natal care” OR AB “pre natal service” OR AB “pre natal services” OR AB “antenatal care” OR AB “antenatal service” OR AB “antenatal services” OR AB “ante natal care” OR AB “ante natal service” OR AB “ante natal services” OR AB “maternal care” OR AB “maternal service” OR AB “maternal services” OR AB childbirth OR AB childbirths | 42,331 |
| 2 | (MH "Health Facilities+")  OR TI “Health Facilit*” OR TI “Medical Cent*”  OR TI “Ambulatory Care Facilit*”  OR TI “Health Cent*” OR TI “Midwifery Servic*” OR TI “Nurse-Midwifery Servic*” OR TI “Nursing Servic*” OR TI “Obstetric Servic*” OR TI “Delivery *Room**”*OR*TI “Nursing Unit*” OR TI “Self-Care Unit*” OR TI “Health Care Facilit*” OR TI “Hospital*”  OR TI “Patients' Room*”  OR  TI “Regional Cent*”  OR TI “facility based” OR AB “Health Facilit*” OR AB “Medical Cent*”  OR AB “Ambulatory Care Facilit*”  OR AB “Health Cent*” OR AB “Midwifery Servic*” OR AB “Nurse-Midwifery Servic*” OR AB “Nursing Servic*” OR AB “Obstetric Servic*” OR AB “Delivery *Room**”*OR*AB “Nursing Unit*” OR AB “Self-Care Unit*” OR AB “Health Care Facilit*” OR AB “Hospital*”  OR AB “Patients' Room*”  OR  AB “Regional Cent*”  OR AB “facility based” | 370,606 |
| 3 | “facility based delivery” OR TI “facility based deliveries” OR TI “facility delivery” OR TI “facility deliveries” OR TI “facility based births” OR TI “facility based birth” OR “facility birth” OR TI “facility births” OR TI “clinic delivery” OR TI “clinic deliveries” OR TI “clinic births” OR TI “clinic birth” OR TI “hospital delivery” OR TI “hospital deliveries” OR TI “hospital birth” OR TI “hospital births” OR TI “hospital childbirth” OR TI “hospital childbirths” OR TI “hospital based deliveries” OR TI “hospital based delivery” OR TI “hospital based births” OR TI “institutional birth” OR TI “institutional births” OR TI “institutional childbirth” OR TI “institutional childbirths” OR TI “institutional delivery” OR TI “institutional deliveries” OR “facility based delivery” OR AB “facility based deliveries” OR AB “facility delivery” OR AB “facility deliveries” OR AB “facility based births” OR AB “facility based birth” OR “facility birth” OR AB “facility births” OR AB “clinic delivery” OR AB “clinic deliveries” OR AB “clinic births” OR AB “clinic birth” OR AB “hospital delivery” OR AB “hospital deliveries” OR AB “hospital birth” OR AB “hospital births” OR AB “hospital childbirth” OR AB “hospital childbirths” OR AB “hospital based deliveries” OR AB “hospital based delivery” OR AB “hospital based births” OR AB “institutional birth” OR AB “institutional births” OR AB “institutional childbirth” OR AB “institutional childbirths” OR AB “institutional delivery” OR AB “institutional deliveries” | 421 |
| 4 | TX "developing country” OR TX ”developing countries” OR TX ”developing nation” OR TX ”developing nations” OR TX ”developing population” OR TX ”developing populations” OR TX ”developing world” OR TX ”less developed country” OR TX ”less developed countries” OR TX ”less developed nation” OR TX ”less developed nations” OR TX ”less developed population” OR TX ”less developed populations” OR TX ”less developed world” OR TX ”lesser developed country” OR TX ”lesser developed countries” OR TX ”lesser developed nation” OR TX ”lesser developed nations” OR TX ”lesser developed population” OR TX ”lesser developed populations” OR TX ”lesser developed world” OR TX ”under developed country” OR TX ”under developed countries” OR TX ”under developed nation” OR TX ”under developed nations” OR TX ”under developed population” OR TX ”under developed populations” OR TX ”under developed world” OR TX ”underdeveloped country” OR TX ”underdeveloped countries” OR TX ”underdeveloped nation” OR TX ”underdeveloped nations” OR TX ”underdeveloped population” OR TX ”underdeveloped populations” OR TX ”underdeveloped world” OR TX ”middle income country” OR TX ”middle income countries” OR TX ”middle income nation” OR TX ”middle income nations” OR TX ”middle income population” OR TX ”middle income populations” OR TX ”low income country” OR TX ”low income countries” OR TX ”low income nation” OR TX ”low income nations” OR TX ”low income population” OR TX ”low income populations” OR TX ”lower income country” OR TX ”lower income countries” OR TX ”lower income nation” OR TX ”lower income nations” OR TX ”lower income population” OR TX ”lower income populations” OR TX ”underserved country” OR TX ”underserved countries” OR TX ”underserved nation” OR TX ”underserved nations” OR TX ”underserved population” OR TX ”underserved populations” OR TX ”underserved world” OR TX ”under served country” OR TX ”under served countries” OR TX ”under served nation” OR TX ”under served nations” OR TX ”under served population” OR TX ”under served populations” OR TX ”under served world” OR TX ”deprived country” OR TX ”deprived countries” OR TX ”deprived nation” OR TX ”deprived nations” OR TX ”deprived population” OR TX ”deprived populations” OR TX ”deprived world” OR TX ”poor country” OR TX ”poor countries” OR TX ”poor nation” OR TX ”poor nations” OR TX ”poor population” OR TX ”poor populations” OR TX ”poor world” OR TX ”poorer country” OR TX ”poorer countries” OR TX ”poorer nation” OR TX ”poorer nations” OR TX ”poorer population” OR TX ”poorer populations” OR TX ”poorer world” OR TX ”developing economy” OR TX ”developing economies” OR TX ”less developed economy” OR TX ”less developed economies” OR TX ”lesser developed economy” OR TX ”lesser developed economies” OR TX ”under developed economy” OR TX ”under developed economies” OR TX ”underdeveloped economy” OR TX ”underdeveloped economies” OR TX ”middle income economy” OR TX ”middle income economies” OR TX ”low income economy” OR TX ”low income economies” OR TX ”lower income economy” OR TX ”lower income economies” OR TX ”low gdp” OR TX ”low gnp” OR TX ”low gross domestic” OR TX ”low gross national” OR TX ”lower gdp” OR TX ”lower gnp” OR TX ”lower gross domestic” OR TX ”lower gross national" OR TX lmic OR TX lmics OR TX "third world” OR TX ”lami country” OR TX ”lami countries” OR TX ”transitional country” OR TX ”transitional countries" | 36,981 |
| 5 | TI Africa OR TI Asia OR TI Caribbean OR TI West Indies OR TI South America OR TI Latin America OR TI Central America OR TI Afghanistan OR TI Albania OR TI Algeria OR TI Angola OR TI Antigua OR TI Barbuda OR TI Argentina OR TI Armenia OR TI Armenian OR TI Aruba OR TI Azerbaijan OR TI Bahrain OR TI Bangladesh OR TI Barbados OR TI Benin OR TI Byelarus OR TI Byelorussian OR TI Belarus OR TI Belorussian OR TI Belorussia OR TI Belize OR TI Bhutan OR TI Bolivia OR TI Bosnia OR TI Herzegovina OR TI Hercegovina OR TI Botswana OR TI Brasil OR TI Brazil OR TI Bulgaria OR TI Burkina Faso OR TI Burkina Fasso OR TI Upper Volta OR TI Burundi OR TI Urundi OR TI Cambodia OR TI Khmer Republic OR TI Kampuchea OR TI Cameroon OR TI Cameroons OR TI Cameron OR TI Camerons OR TI Cape Verde OR TI Central African Republic OR TI Chad OR TI Chile OR TI China OR TI Colombia OR TI Comoros OR TI Comoro Islands OR TI Comores OR TI Mayotte OR TI Congo OR TI Zaire OR TI Costa Rica OR TI Cote d'Ivoire OR TI Ivory Coast OR TI Croatia OR TI Cuba OR TI Cyprus OR TI Czechoslovakia OR TI Czech Republic OR TI Slovakia OR TI Slovak Republic OR TI Djibouti OR TI French Somaliland OR TI Dominica OR TI Dominican Republic OR TI East Timor OR TI East Timur OR TI Timor Leste OR TI Ecuador OR TI Egypt OR TI United Arab Republic OR TI El Salvador OR TI Eritrea OR TI Estonia OR TI Ethiopia OR TI Fiji OR TI Gabon OR TI Gabonese Republic OR TI Gambia OR TI Gaza OR TI Georgia Republic OR TI Georgian Republic OR TI Ghana OR TI Gold Coast OR TI Greece OR TI Grenada OR TI Guatemala OR TI Guinea OR TI Guam OR TI Guiana OR TI Guyana OR TI Haiti OR TI Honduras OR TI Hungary OR TI India OR TI Maldives OR TI Indonesia OR TI Iran OR TI Iraq OR TI Isle of Man OR TI Jamaica OR TI Jordan OR TI Kazakhstan OR TI Kazakh OR TI Kenya OR TI Kiribati OR TI Korea OR TI Kosovo OR TI Kyrgyzstan OR TI Kirghizia OR TI Kyrgyz Republic OR TI Kirghiz OR TI Kirgizstan OR TI "Lao PDR" OR TI Laos OR TI Latvia OR TI Lebanon OR TI Lesotho OR TI Basutoland OR TI Liberia OR TI Libya OR TI Lithuania OR TI Macedonia OR TI Madagascar OR TI Malagasy Republic OR TI Malaysia OR TI Malaya OR TI Malay OR TI Sabah OR TI Sarawak OR TI Malawi OR TI Nyasaland OR TI Mali OR TI Malta OR TI Marshall Islands OR TI Mauritania OR TI Mauritius OR TI Agalega Islands OR TI Mexico OR TI Micronesia OR TI Middle East OR TI Moldova OR TI Moldovia OR TI Moldovian OR TI Mongolia OR TI Montenegro OR TI Morocco OR TI Ifni OR TI Mozambique OR TI Myanmar OR TI Myanma OR TI Burma OR TI Namibia OR TI Nepal OR TI Netherlands Antilles OR TI New Caledonia OR TI Nicaragua OR TI Niger OR TI Nigeria OR TI Northern Mariana Islands OR TI Oman OR TI Muscat OR TI Pakistan OR TI Palau OR TI Palestine OR TI Panama OR TI Paraguay OR TI Peru OR TI Philippines OR TI Philipines OR TI Phillipines OR TI Phillippines OR TI Poland OR TI Portugal OR TI Puerto Rico OR TI Romania OR TI Rumania OR TI Roumania OR TI Russia OR TI Russian OR TI Rwanda OR TI Ruanda OR TI Saint Kitts OR TI St Kitts OR TI Nevis OR TI Saint Lucia OR TI St Lucia OR TI Saint Vincent OR TI St Vincent OR TI Grenadines OR TI Samoa OR TI Samoan Islands OR TI Navigator Island OR TI Navigator Islands OR TI Sao Tome OR TI Saudi Arabia OR TI Senegal OR TI Serbia OR TI Montenegro OR TI Seychelles OR TI Sierra Leone OR TI Slovenia OR TI Sri Lanka OR TI Ceylon OR TI Solomon Islands OR TI Somalia OR TI Sudan OR TI Suriname OR TI Surinam OR TI Swaziland OR TI Syria OR TI Tajikistan OR TI Tadzhikistan OR TI Tadjikistan OR TI Tadzhik OR TI Tanzania OR TI Thailand OR TI Togo OR TI Togolese Republic OR TI Tonga OR TI Trinidad OR TI Tobago OR TI Tunisia OR TI Turkey OR TI Turkmenistan OR TI Turkmen OR TI Uganda OR TI Ukraine OR TI Uruguay OR TI USSR OR TI Soviet Union OR TI Union of Soviet Socialist Republics OR TI Uzbekistan OR TI Uzbek OR Vanuatu OR TI New Hebrides OR TI Venezuela OR TI Vietnam OR TI Viet Nam OR TI West Bank OR TI Yemen OR TI Yugoslavia OR TI Zambia OR TI Zimbabwe OR TI Rhodesia OR AB Africa OR AB Asia OR AB Caribbean OR AB West Indies OR AB South America OR AB Latin America OR AB Central America OR AB Afghanistan OR AB Albania OR AB Algeria OR AB Angola OR AB Antigua OR AB Barbuda OR AB Argentina OR AB Armenia OR AB Armenian OR AB Aruba OR AB Azerbaijan OR AB Bahrain OR AB Bangladesh OR AB Barbados OR AB Benin OR AB Byelarus OR AB Byelorussian OR AB Belarus OR AB Belorussian OR AB Belorussia OR AB Belize OR AB Bhutan OR AB Bolivia OR AB Bosnia OR AB Herzegovina OR AB Hercegovina OR AB Botswana OR AB Brasil OR AB Brazil OR AB Bulgaria OR AB Burkina Faso OR AB Burkina Fasso OR AB Upper Volta OR AB Burundi OR AB Urundi OR AB Cambodia OR AB Khmer Republic OR AB Kampuchea OR AB Cameroon OR AB Cameroons OR AB Cameron OR AB Camerons OR AB Cape Verde OR AB Central African Republic OR AB Chad OR AB Chile OR AB China OR AB Colombia OR AB Comoros OR AB Comoro Islands OR AB Comores OR AB Mayotte OR AB Congo OR AB Zaire OR AB Costa Rica OR AB Cote d'Ivoire OR AB Ivory Coast OR AB Croatia OR AB Cuba OR AB Cyprus OR AB Czechoslovakia OR AB Czech Republic OR AB Slovakia OR AB Slovak Republic OR AB Djibouti OR AB French Somaliland OR AB Dominica OR AB Dominican Republic OR AB East Timor OR AB East Timur OR AB Timor Leste OR AB Ecuador OR AB Egypt OR AB United Arab Republic OR AB El Salvador OR AB Eritrea OR AB Estonia OR AB Ethiopia OR AB Fiji OR AB Gabon OR AB Gabonese Republic OR AB Gambia OR AB Gaza OR AB Georgia Republic OR AB Georgian Republic OR AB Ghana OR AB Gold Coast OR AB Greece OR AB Grenada OR AB Guatemala OR AB Guinea OR AB Guam OR AB Guiana OR AB Guyana OR AB Haiti OR AB Honduras OR AB Hungary OR AB India OR AB Maldives OR AB Indonesia OR AB Iran OR AB Iraq OR AB Isle of Man OR AB Jamaica OR AB Jordan OR AB Kazakhstan OR AB Kazakh OR AB Kenya OR AB Kiribati OR AB Korea OR AB Kosovo OR AB Kyrgyzstan OR AB Kirghizia OR AB Kyrgyz Republic OR AB Kirghiz OR AB Kirgizstan OR AB "Lao PDR" OR AB Laos OR AB Latvia OR AB Lebanon OR AB Lesotho OR AB Basutoland OR AB Liberia OR AB Libya OR AB Lithuania OR AB Macedonia OR AB Madagascar OR AB Malagasy Republic OR AB Malaysia OR AB Malaya OR AB Malay OR AB Sabah OR AB Sarawak OR AB Malawi OR AB Nyasaland OR AB Mali OR AB Malta OR AB Marshall Islands OR AB Mauritania OR AB Mauritius OR AB Agalega Islands OR AB Mexico OR AB Micronesia OR AB Middle East OR AB Moldova OR AB Moldovia OR AB Moldovian OR AB Mongolia OR AB Montenegro OR AB Morocco OR AB Ifni OR AB Mozambique OR AB Myanmar OR AB Myanma OR AB Burma OR AB Namibia OR AB Nepal OR AB Netherlands Antilles OR AB New Caledonia OR AB Nicaragua OR AB Niger OR AB Nigeria OR AB Northern Mariana Islands OR AB Oman OR AB Muscat OR AB Pakistan OR AB Palau OR AB Palestine OR AB Panama OR AB Paraguay OR AB Peru OR AB Philippines OR AB Philipines OR AB Phillipines OR AB Phillippines OR AB Poland OR AB Portugal OR AB Puerto Rico OR AB Romania OR AB Rumania OR AB Roumania OR AB Russia OR AB Russian OR AB Rwanda OR AB Ruanda OR AB Saint Kitts OR AB St Kitts OR AB Nevis OR AB Saint Lucia OR AB St Lucia OR AB Saint Vincent OR AB St Vincent OR AB Grenadines OR AB Samoa OR AB Samoan Islands OR AB Navigator Island OR AB Navigator Islands OR AB Sao Tome OR AB Saudi Arabia OR AB Senegal OR AB Serbia OR AB Montenegro OR AB Seychelles OR AB Sierra Leone OR AB Slovenia OR AB Sri Lanka OR AB Ceylon OR AB Solomon Islands OR AB Somalia OR AB Sudan OR AB Suriname OR AB Surinam OR AB Swaziland OR AB Syria OR AB Tajikistan OR AB Tadzhikistan OR AB Tadjikistan OR AB Tadzhik OR AB Tanzania OR AB Thailand OR AB Togo OR AB Togolese Republic OR AB Tonga OR AB Trinidad OR AB Tobago OR AB Tunisia OR AB Turkey OR AB Turkmenistan OR AB Turkmen OR AB Uganda OR AB Ukraine OR AB Uruguay OR AB USSR OR AB Soviet Union OR AB Union of Soviet Socialist Republics OR AB Uzbekistan OR AB Uzbek OR Vanuatu OR AB New Hebrides OR AB Venezuela OR AB Vietnam OR AB Viet Nam OR AB West Bank OR AB Yemen OR AB Yugoslavia OR AB Zambia OR AB Zimbabwe OR AB Rhodesia | 92,352 |
| 6 | (MH "Africa+") OR (MH "Asia+") OR (MH "South America+") OR (MH "Central America+") OR (MH "Mexico+") OR (MH "West Indies+") OR (MH "Atlantic Islands+") OR (MH "Armenia") OR (MH "Azerbaijan") OR (MH "Europe, Eastern") OR (MH "Georgia (Republic)") OR (MH "Indian Ocean Islands") OR (MH "Pacific Islands") OR (MH "Armenia") OR (MH "Azerbaijan") OR (MH "Europe, Eastern") OR (MH "Georgia (Republic)") OR (MH "Indian Ocean Islands") OR (MH "Pacific Islands") | 179,818 |
| 7 | (MH "cluster sample+") or TX life experiences or TX human science or TX discourse* analysis or TX narrative analysis or TX lived experience* or TX field research or TX field studies or TX field study or TX giorgi* or TX husserl* or TX merleau ponty* or TX van kaam* or TX van manen* or TX spiegelberg* or TX colaizzi* or TX heidegger* or TX participant observ* or TX data saturat* or TX semiotics or TX heuristic or TX hermeneutic* or TX etic or TX emic or TX focus group* or TX purpos* sampl* or TX constant comparison or TX constant comparative or TX grounded research or TX grounded studies or TX grounded study or TX grounded theor* or TX phenomenol* or TX ethnon* or TX qualitative or (MH "ethnological research") or (MH "ethnography") or (MH "phenomenology") or (MH "focus groups") or (MH "discourse analysis") or (MH "theoretical sample") or (MH "field studies") or (MH "constant comparative method") or (MH "thematic analysis") or (MH "content analysis") or (MH "observational methods+") or (MH "purposive sample") or (MH "qualitative validity+") or (MH "grounded theory") or (MH “action research”) or (MH “naturalistic inquiry”) or (MH "ethnonursing research") or (MH "phenomenological research") or (MH "ethnographic research") or (MH "qualitative studies") or (MH "Interviews+") or (MH "Narratives") or (MH "Videorecording+") or (MH "Audiorecording") or (MH "Historical Records") | 284,524 |
| 8 | ((S1 AND S2) OR S3) AND (S4 OR S5 OR S6) AND S7 | 867 |

**Appendix C: Codebook**

| **Code** | **Description** | **Illustrative quote** |
| --- | --- | --- |
| **1.0 Cost** | | |
| **1.1 Direct costs associated with facility birth** | This code refers to the cost of the delivery process, including:  - cost of delivery - process for obtaining funds to pay for delivery services (loans, family, friends, selling land, etc.) - acceptability of paying for services in the case of a complication | *“We mortgaged our land. The mortgager will take all the crops off that land … Now we will not get the crop from that land. The whole year we will have to buy the crops from the market.” (ID 273, poverty score 19) (8:216)   ‘We did not have the money to go to the hospital, so I had to deliver the child at home,’ (Sakhwai, Mumbai). (12:354)* |
| **1.2 Additional costs associated with facility birth** | This code refers to costs associated with facility birth, in addition to the direct cost of delivery. Examples include: - medicine - blood  - lab tests - accommodation/meals for family/ friends accompanying birth - bribes paid for better service - admission fees - payment to informal workers (ward boys or ayas) | *“My husband had to spend so much money on meals. Every day we spent about BDT 200 for our relatives coming from the village. I stayed three days in hospital and the meal costs exceeded BDT 1000.” (Rownak) (11:176)   “At first I took 1200 BDT, then again came home and took the rest of the money with me. I bought the medicine on credit. I deposited 1200 BDT to the pharmacy and bought all the medicine and brought it to the hospital. At 5pm after handover of the medicines I came back to our house to take the money and then pay back … we borrowed the money from our friends and relatives.” (ID 114, poverty score 26) (8:216)* |
| **1.3 Cost influencing type of facility** | Costs of facility birth at different levels of facility may influence if a woman delivers at a government vs. private, ayurvedic vs. allopathic, dispensary vs. hospital. | *“My sister lost her baby while giving birth because her husband had no money, he was not cooperative. She asked him to come to the hospital, he said; 'no, you will be at the dispensary'. At the dispensary they failed. Then when they told him to find a transport for her to come to the hospital, he said he had no money, so he left her there, went home. (…) and she ended with a dead baby” (KRHUser2). (27:123)* |
| **2.0 Influence of others on birthing decisions** | | |
| **2.1 Husbands/male partners** | This code refers to the involvement of husbands or male partners on birth location decisions, including the influence that they have on the decision and any support that they provide that would facilitate a facility birth. | *‘If I get sick, my husband intends to take me to the HF. I told him that I wanted to give birth at home, because I was afraid of HFs because of the instruments. Then he encouraged and persuaded me, then they took me to the HF. I gave birth in a nice situation, now I understand HF is good’. (Woman who delivered at health facility - FGD 3) (15:9)* |
| **2.2 Mother/Mother-in-law/elderly women** | This code refers to the involvement of elderly women (mothers, mother-in-laws, etc.) on birth location decisions. | *The grandmother, she just tells the granddaughter: - don't worry, deliver here so that I can take care of you. So the girl listens more to the grandmother than she remembers what was said at the clinic." (21:72)* |
| **2.3 Interplay between many actors** | This code refers to the complicated interplay of many actors in the decision about birth location. | *Women expressed that accessing ANC was woman’s decision; however, regarding delivery care the decision making was much more dependent on others. Women reported that husbands were in favor of institutional delivery, whereas, the elderly favored home delivery. Women also revealed that when emergency care was sought, community members called HEWs. When HEWs were called, they became in charge, since they are responsible and accountable for any health event encountered at the community level. (15:10)* |
| **2.4 Personnel link to health facility** | This code refers personal links to staff at health facilities, for example a family member or friend works at a health facility or an untrained provider facilitates a connection to his associated health facility | *It was observed that the unqualified ‘village doctors’ often have links to health facilities. In one case, a woman reported that the village doctor arranged deliveries for women in a public facility for some payment (IDI#BG19), while, in another case, a women explained how the village doctors actually owned a private health clinic which employed professional medical staff (IDI#BG18). In cases where these non-qualified practitioners have financial links to professional facilities, they may play an active role in encouraging women to seek professional care—although in cases where women were referred to private facilities, they faced much higher costs compared to those in public facilities. (30:442)* |
| **3.0 Plan for childbirth** | This code refers to any plans or lack of plans that a woman or her family make for her delivery. | *IDI#UG14—20 years old, local religion, second pregnancy, some primary education: “My husband would hire a [small] motorcycle to take me for antenatal care or sometimes he would take me on his bicycle. My husband's friend would sometimes tell us to put fuel in the motorcycle, and he would take me to the health centre. At nine months, I bought petrol and kept it in the house. It is the [fuel] we put in my husband's friend's motorcycle to [reach] the health centre.”(30:442)   Many interviewees articulated that because labor is unpredictable, planning transportation and child care in preparation for arriving at a health service is difficult. One woman said, "“You don’t know when the pain’s going to hit you . . . what can we do?” (1:49)"* |
| **4.0 HIV** | | |
| **4.1 Fear of HIV testing at a facility** | This code refers to the fear that some women have that they will have compulsory HIV testing at delivery, which may result in violence from their partner or have them forced to learn their status when they may not want to. | *If you go to the hospital to deliver, they will insist that you undergo HIV testing and many of us are not ready for that. If you go there and find out that you are positive, what will you do?” (19:587)* |
| **4.2 Fear of HIV+ disclosure and discrimination** | This code refers to the fear that some women have that their HIV status will be disclosed in a health facility and that HCWs and other women will discriminate against them. | *“This problem of a mother being HIV-positive and the husband is not aware is a problem to us because it is really difficult to start telling the husband, ‘Your wife has come to deliver here and she is positive. We have given her medicine.’ It becomes difficult to explain … When she is asked she says, ‘My husband is not aware and I don't want him to know.’ So it becomes difficult for us to explain to the husbands because if you say it maybe the husband can react, even might decide to leave her here.” (Nurse, maternity unit, health center) (22:942)* |
| **5.0 Transportation/access** | | |
| **5.1 Proximity of health facility** | This code refers to the perception of the distance and time to a health facility and whether this perception of proximity is a facilitator or barrier | *Maybe distance is an obstacle in addition to the costs. You need to ride a motorcycle to reach the health facility. So people prefer having the health professional to come to their houses, especially at night time when it is hard to get transport. (In-depth interview with the head of health centre, Garut) (4:8)* |
| **5.2 Vehicle availability** | This code refers to the availably of vehicles when women need to access a health facility. | *“I had to run to the neighbour to see if I could get a bicycle. But he was not around, so I had to run more and I saw my friend with a motorcycle, I requested him to take my wife but he said; 'I have not enough fuel to Kashozi'. I had to return back, I was afraid thinking; 'my wife is going to die'. I haven't seen her giving birth at home. So, while running I met with another friend who had a bicycle and I requested the bicycle. He said; 'I am running to the shop and then I will be back in few minutes'. When I came home I found my wife was just trying to push, I just passed the eye and saw something swelling. I didn't wait to see if it was the baby or not, I had to run, thinking where to run to the neighbour thinking that; 'my wife is dying', thinking of the mother who can help, one woman nearby, I had to run to her” (BHDUser4Husband). (27:123)* |
| **5.3 Out-of-hours availability** | This code refers to the availability of health facilities and transportation in off-hours, such as nights and weekends | *As it is known, labor often comes during the night. People coming from rural villages pay a lot of money for transportation. However, when we reach there, it is possible that the facility is closed. Even if they are open, medications and equipment are often in short supply. Eventually, we end up taking prescriptions to buy from private pharmacies. Some health workers who are on night duty are also not competent enough to manage deliveries. Many times, they refer all laboring mothers to other hospitals. We face similar problems during weekends. These are serious problems which discourage us from going to a health facility in the first place. Male FGD participant 1(27:6)* |
| **5.4 Referrals** | This code refers to the issues that women have in receiving and accessing referrals. | *It was difficult and time consuming to organise emergency referral outside district capitals. For example, during fieldwork in Manufahi district a health post midwife was assisting a home birth and needed to refer the labouring woman. Lacking any transport or communication she sent a family member to catch a bus to Same, the district capital, with a folded note which read: "Emergency!!!! Please send an ambulance because there is a patient who will give birth. Labour began yesterday. She is bleeding often. Because it is too far (Tukunu) I request the hospital to come and pick her up. – Health post midwife, Manufahi district (3:2042)"* |
| **6.0 Policies** | This code refers to macro level policies that may influence the decision to deliver in a health facility. Includes instances where policies or programs may not actually be facilitating facility delivery due to poor communication or understanding of the program. | *There are so many people seeking care from traditional birth attendants. We have already explained that if you have Jamkesmas, you do not have to pay anything to use midwives' services. But then they said they were still afraid that they would be required to pay. One day they also said they were ashamed of using the midwives services without paying anything. (In-depth interview with a cadre, Sukarame, Garut) (4:6-7)    People were afraid that they will be neglected [by a health provider] if they used Jamkesmas. (In-depth interview with a community leader, Panyutran, Ciamis) (4:7)* |
| **7.0 Perception of risk** | | |
| **7.1 HCWs views on community risk perception** | This code refers to the HCWs views of how the community perceives their risks during the childbirth period. | *“We are not big enough to attack the community, so that we talk and they understand” (BHFHP5). (27:125)* |
| **7.2 User awareness and identification of risks associated with childbirth** | This code refers to users' awareness and identification of risks that may be associated with childbirth. | *“After that there is the so called “obbaatii” (placenta), which keeps the baby when she is in the abdomen. That is a problem just like giving birth. If not lucky, it can even force us to go to the health facilities” (woman, home birth – 40 yrs.). (29:27)* |
| **7.3 Individual risk perception facilitating facility delivery** | This code refers to an individual's personal risk perception in a pregnancy and how that may or may not influence their choice of birth location | *IDI#UG10—20 years old, Catholic, first pregnancy, secondary education: “I also prefer going to Health Centre A because it has a doctor, you never know, in the case of a problem, one can be sure of a doctor's help.” (441)* |
| **7.4 Previous birth facilitating future facility birth** | This code refers to situations where a previous positive experience with a health facility delivery may facilitate a future health facility delivery or where a previous home birth experience may facilitate a future health facility delivery. | *When I started to go into labour I came here straight away because I already had experience from last time. Last time I tried to birth at home and I had problems with the baby. – Group discussion, Same birth centre (3:2040)* |
| **7.5 Previous birth NOT facilitating future facility birth** | This code refers to situations where a previous birth experience did NOT facilitate a future health facility birth. For example, a positive experience with home birth or uncomplicated previous facility birth encouraged a woman to deliver at home. | *“My first baby was delivered in hospital, it went smoothly. Besides, I had two antenatal check-ups for this one, and everything looked good. Also I didn't have the money, so I did not go to hospital this time.” (Participant 29, age 33, second birth) (2:41)* |
| **7.6 Link to HF from previous care** | This code refers to links to HF fostered through ANC visits (both as a facilitator and not as a facilitator), previous births, previous interactions with the health system, etc. | *“Any woman could deliver safely anywhere.” (19:586) Unless a woman has been told during antenatal care to expect a difficult delivery, it would be irrational for her to still seek hospital-based birthing after faithfully attending antenatal care. You will just go there and waste time and money. We take antenatal seriously because most of us will not go back to deliver in the hospital….(19:586)* |
| **8.0 Perceived quality of care** | | |
| **8.1 Patient-provider interactions** | This code refers to both positive and negative experience with the bedside manner of HCWs. Included in this code: - rude or inconsiderate treatment by HCWs - HCW abuse of patients - kind treatment of patients by HCWs | *Jamila: Those dāīs in the government hospital—the ones who deliver babies—asked Razia how many children she had. I don’t know if Razia said 8 or 9, but on hearing this, the nurse slapped her face saying “you have so many children and even so you are busy making more.” (Authors’ field notes 9 February 2004) (5:1715)* |
| **8.2 Health facility infrastructure** | This code refers to the different aspects of the infrastructure (+ and -) of a HF that may affect an individual's perception of the quality of the HF. Included in this code:  - HF supplies (blood, machinery, drugs, etc.) - HF personnel (availability, coverage) - Available space in the HF (waiting area, separate maternity area) | *“They [public health facilities] don’t have the equipment to treat patients. They lack equipment to check status of pregnancy and other signs of complications”- [adult female discussant]. (26:6)   ‘Razia’s attendants deemed her situation serious only when the baby’s arm appeared. With no one at the PHC responsible for delivering the baby or referring her elsewhere, she was taken to the government women’s hospital in Bijnor town on a tractor-trolley requisitioned from a neighbour. (5:1714)* |
| **8.3 Waiting, neglect & lack of communication** | This code refers to waiting times to receive care within a facility, perception of HCW neglect of patient needs, and lack of communication between HCWs and women/families | *“It has been reported that maternal deaths happen even in the hospital. So it is not because the mothers are attended by the TBA [that women die]. They don't receive enough care at the hospital. The health providers they are not always close to the mother, the mother may request to be examined, but they just say; 'it is not the time for you, it is not yet'. She may bleed without anybody noticing it. But the TBA is always close to her, when she is requesting to come and see she is always available close-by, she may detect any changes that occur. I hear people talking that many women die at the hospital. Especially those who are not well experienced in service, the health providers, when they are called, they say; 'the time is not ready to push. Don't disturb me; we were not together when you conceived'” (BHDUser9). (27:122)* |
| **8.4 Inequities of care** | This code refers to the inequities in healthcare provision due to an individual's SES. For example, this includes the user's perception that seeking care at a HF as a poor woman would lead to mistreatment or refusal to provide care. | *Martha (aged 34) also noted, “It costs a lot to deliver in the hospital and when poor people like us go there, [we] are treated shoddily.” (19:587)* |
| **8.5 Medical treatment by HCWs** | This code refers to the perceived quality of medical treatment provided by skilled and unskilled providers, including the following 4 domains: - positive perceptions of medical treatment provided by skilled HCWs - negative perceptions of medical treatment provided by skilled HCWs - positive perceptions of medical treatment provided by TBAs - negative perceptions of medical treatment provided by TBAs | *“Being in the hospital, when I take my wife there, I am very, very satisfied of the care she is receiving. I don't feel afraid when the mother is with the staff within the hospital. Because in case of any problems they easily detect and they know much concerning conducting deliveries” (BHDUser4Husband) (27:124)   I had repeated antenatal visits during my first pregnancy. In one of my visits (at the ninth month of pregnancy), I experienced a severe crampy abdominal pain for which the health care provider advised me to take medications assuming it is caused by intestinal worms. Lately, I realized that I was actually in true labor and was thus forced to have my first child delivered at home. That experience eroded my confidence and trust on health professionals’ competence as a result of which I decided to deliver at home for all of my subsequent children. Female FGD participant 1 (27:6)* |
| **9.0 Medicalization of childbirth** | | |
| **9.1 Birth is a natural event** | This code refers to the conceptualization that birth is a naturally occurring event in a woman's life and therefore does not require any medical interventions. If complications arise, then HF delivery is considered acceptable. | *“Everybody is born at home, and it seems natural to keep on doing that” (elderly woman – 56 yrs.). (29:25)* |
| **9.2 Supportive attendance at birth** | This code refers to the desire to have family and friends accompany the woman in the labor ward at a facility. | *Being with your family you can explain any pain you have to them, your relatives are there to give you encouragements, being alone with a nurse in case of complications during delivery it will be difficult to handle. Your relatives are there to take care, being with them you feel comfortable (BHFUser1sisterinlaw) (Sorensen et al., 2011)* |
| **9.3 Unfamiliar/undesirable birth practices** | This code refers to any unfamiliar birth practices that may occur in a HF delivery, compared to a home delivery. This includes: - birthing position - "excess" vaginal exams" | *I can't even think about giving birth lying down on the bed. How is it possible? How do women push down in this position? I don't think I would be able to deliver at the BHC!' (10:83)* |
| **9.4 Privacy** | This code refers to the lack of privacy in a HF delivery, including exposing private parts and people watching the delivery. | *“In the hospital, everyone looks at us and they open our legs.” (1:49)* |
| **9.5 Fear of cutting (episiotomy/CS)** | This code refers to the fear of cutting that may occur in a HF delivery, including episiotomy and CS. There are two subcategories in this code: - perceived unjustified cutting by HCWs - fear of the actual cut | *"A woman is born to deliver vaginally" (21:72)   “In hospitals, doctors are ready for Caesarean, if the child birth lasts only for a short longer time; they will soon precede an operation” [Mother 2, 7, 15].   “After referring women to hospitals, physicians hurry in decision making; and most of the time they offer women Caesarean sections” [Midwife 4]. (14:5)* |
| **10.0 Intersection of traditionalism and modernity** | | |
| **10.1 Influence of tradition & culture** | This code refers to the role of faith and tradition in determining the location of delivery | *“God decides because everything is destined; if when I am pregnant I happen to move from one place to another, the place where I have labour pain and eventually deliver is the place God has decided I would deliver”- [adult female discussant]. (26:3)* |
| **10.2 Modernity** | This code refers to the desire to appear modern facilitating HF delivery, including: - home delivery associated with old age and no education - modern acceptance of HF interventions | *I: What if the delivery is done at home? R: I suppose home is preferred by the elderly people of old day. In this consideration, it is better not to keep the expectant [mother] at home. I: What are the problems that can be faced at home? R: It is not possible to do everything at home. Women who are ignorant and not up-to-date think that the delivery is always done normally. It is foolish if we don’t keep pace with the modern age. In present circumstances, it is better to contact the doctor. A nurse is needed to stay beside the patient all the time in order to look after her meals and medicine. - 26-year old husband, CSBA home delivery (13:8)* |
| **10.3 Delay in transition from unskilled to skilled care** | This code refers to the delay in seeking care from a skilled provider due:- seeking care from an unskilled provider first- TBA lack of recognition of incompetence- existence of medical pluralism - women seek advice from many sources | *There are many cases like this, for example an obstructed labour with excessive bleeding, or retained placenta cases. They just wait until the traditional birth attendants could no longer manage it. Sometimes we arrived late and the mother already had severe oedema and was in a very weak condition. (In-depth interview with a health centre midwife, Ciamis) (4:7)* |
| **10.4 Cooperation between informal and formal health systems** | This code refers to dangerous situations that arise when TBAs are not integrated into the health system, or TBAs don't want to cooperate with the health system. It also refers to the pragmatic decision making women tend to use, including the belief that it is not a contradiction to use both traditional and medical providers | *“If midwives refer a woman to the hospital, the woman and her family think she is not competent, and she will lose all credibility in the eyes of the patient” [Midwife 4, 8]. (14:6)* |
| **10.5 Seeking unskilled care first** | This code refers to women who seek care from an unskilled or traditional provider prior to seeking care from a skilled or medical provider. | *If my wife goes into labor, the first thing I would do is call a traditional birth attendant. If she (traditional birth attendant) believes that the labor can be managed at home, we will stay at home. We will go to a health center only if the traditional birth attendant says so. We have confidence in them. Hence I comply with whatever the traditional birth attendant tells me to do to save the life of my wife. Male FGD participant 3 (27:5)* |
| **11.0 Logistics of home birth** | This code refers to the perception that home births are logistically easier. For example, a woman can maintain her domestic responsibilities if she delivers at home and she won't have to arrange accommodation for those who accompany her birth | *They just said they do not want to bother anyone. Delivery in the midwife's place means someone needs to go and accompany you. At home they can just wait for the delivery time while doing some household chores. (In-depth interview with a cadre, Sukarame, Garut) (4:8)* |

**Appendix F: Study summaries**

**Note:** summaries of included studies that utilized both quantitative and qualitative research methodologies only specify the qualitative components as the quantitative data was not extracted for the purposes of this review. Abbreviations: in-depth interview (IDI), focus group discussion (FGD), participant observation (PO), traditional birth attendant (TBA), community health worker (CHW). (*) Study number does not refer to the number on the reference list.

| Study # * | Study (author/ year) | Location | Sample characteristics | Data collection & analysis | Findings  ** relevant sections summarized by the reviewer* | Quality assessment |
| --- | --- | --- | --- | --- | --- | --- |
| 1 | Otis, K & Brett J (2008) | Yapacani, Santa Cruz, Bolivia | 62 participants presented in findings (44 mothers, 18 fathers) | IDI, PO  Grounded theory, thematic analysis | Respondents generally preferred facility delivery to home delivery, but many families who desired facility delivery were unable to access care. Five main themes were identified to explain the low-rate of facility delivery: (1) fear of or embarrassment to receive care at a public health center; (2) perceived low quality of care at the facility, including unfamiliar birth practices; (3) far distance or other geographical barriers; (4) financial constraints related to transportation or costs of services at facility, including indirect costs; and (5) expectation of an "easy" birth. | High quality |
| 2 | Gao Y; Barclay L; Kildea S; Hao M; & Belton S (2010) | Shanxi province, China | 30 women aged 21-39 years | IDI  Content analysis, thematic analysis | Respondents identified six main barriers to facility birth: (1) financial constraints at the household level; (2) transportation issues; (3) perceived low quality of care at the facility, including dissatisfaction with previous experiences; (4) perceived "easy" birth; (5) preference for TBAs despite their illegality; and (6) high cost of facility delivery, despite government subsidies. The illegality of more than 1 birth was not considered a barrier by the respondents. | Low quality |
| 3 | Wild K; Barclay L; Kelly P; Martins N (2010) | Timor-Leste | 54 participants presented in findings (16 pregnant/ recently delivered women, 5 husbands, 11 FGDs with 33 participants total (women, husbands, and grandmothers) | IDI, FGD  Thematic analysis | Utilization of facilities for delivery was impacted by an individual's experiences (previous birth experiences and perception of risk), social constructs (disease etymology, cultural relevance of traditional treatments, social support, and intergenerational continuity), political factors (decision-making power, SES, and transport infrastructure), and health system factors (location of facilities, referral infrastructure, condition of facilities, perceived treatment by HCWs, and facility policies). | High quality |
| 4 | Titaley C; Hunter C; Dibley M; Heywood P (2010) | West Java Province, Indonesia | 295 participants total (119 mothers, 40 fathers, 26 health professionals, 20 CHWs, 37 TBAs, 42 community and religious leaders, 11 health office staff | IDI, FGD  Content analysis, thematic analysis | Reasons for using TBAs included: (1) economic and pragmatic reasons; (2) trust; (3) intergenerational continuity; (4) perceived risk of delivery; (5) inaccessibly services at the facility; and (6) high perceived quality of care from TBAs. Reasons for home delivery were similar, and included: (1) lower cost than facility delivery; (2) birth viewed as a non-medical event; (3) inaccessible facility services; and (4) convenience of home birth. The primary reason for utilizing faculty-based care was delivery complications. | High quality |
| 5 | Jeffery P & Jeffery R (2010) | Uttar Pradesh, India | Does not provide the exact number of participants, who included mothers, husbands, neighbors, TBAs, and health workers. | IDI, FGD  Thematic analysis | Factors affecting the access and utilization of facility delivery included: (1) facility delivery perceived as unnecessary; (2) distrust of health providers; (3) gaps in provision of services at the facility; (4) low SES; (5) cost of facility-based delivery; and (6) perceived poor treatment and abuse at the facility. | Medium quality |
| 6 | Iyengar S; Iyengar K; Martines J; Dashora K; Deora K (2008) | Udaipur, India | 57 IDI participants (18 women, 39 key informants)  10 FGDs (8-12 mothers, TBAs, and grandmothers each) | IDI, FGD, free listing, rating & ranking  Analysis method not specified by author | Factors affecting the access and utilization of facility delivery included: (1) preference for home birth; (2) preference for and value of TBA; (3) dislike the "hands-off" approach to care in the facility; (4) acceptance of facility-delivery for adolescent mothers or during long or complicated deliveries; (5) decision-making responsibility lies with the father or brother of the parturient woman. | Medium quality |
| 7 | Hadwiger M & Hadwiger S (2012) | Iloilo province, Philippines | 26 women | IDI  Content analysis, thematic analysis | Women preferred home birth to facility birth for the following reasons: (1) trust in God for a safe delivery at home; (2) perception of high facility costs; (3) ability to pay TBAs in installments; (4) home birth is more comfortable (5) perception that health workers are disrespectful; and (6) lack of privacy in the facility. | Medium quality |
| 8 | Pitchforth E; van Teijlingen E; Graham W; Dixon-Woods M; Chowdhury M (2006) | Dhaka, Bangladesh | 44 participants total (25 women, 19 health staff) | IDI, PO  Constant comparison, thematic analysis | Women faced significant financial and social constraints even after reaching a facility for delivery, including: (1) additional costs associated with facility delivery; (2) lack of savings or preparation to pay hospital bills; (3) treatment delays while waiting for funds; (4) drastic measures to obtain cash - mortgage property or borrow from neighbors; and (5) Social Welfare Organization and the "poor fund" are informal mechanisms to assist in treatment costs, but do not function efficiently. | Medium quality |
| 9 | Parkhurst J & Rahman S (2007) | Jhenaidah, Bangladesh | 30 women aged 16-35 years | IDI  Thematic analysis | Women recognized the importance of medical care in an emergency, but most women attempted to deliver at home first and only attended a faculty in case of a complication. Women who attended facilities and received a caesarean section were either referred by a village practitioner, ANC provider, or referred from another facility. High costs associated with caesarean sections led women to avoid them. Women and their families distrusted doctors who recommended caesarean sections, often viewing the operation as unnecessary and would go to extreme measures to avoid them. | Medium quality |
| 10 | Afsana K & Rashid S (2001) | Bangladesh | 25 IDI participants (20 women, 5 TBAs)  3 FGDs (21 women in total)  4 birth observations  2 informal discussions with 4 physicians and 7 paramedics | IDI, FGD, PO  Analysis method not specified by author | Most women preferred to deliver at home and only attended a facility for delivery if complications arose. Decisions-making on location of delivery is hierarchical and men are ultimately responsible. TBAs are viewed as inexpensive compared to facilities, and financial constraints play an important role in accessing facility delivery. Even though government services are free, hidden costs inhibit attendance. Women fear facilities and the risk of an operation, which is considered stigmatizing. Health workers in facilities do not communicate well with the women or her family, forced women to deliver in unfamiliar birth positions, and did not maintain privacy. Any compassionate care provided by the BHC worker was highly regarded, but women perceived health workers to be rude, unhelpful, and dismissive. | High quality |
| 11 | Afsana K (2004) | Bangladesh | 170 participants total (women, husbands, in-laws, TBAs, health workers, health staff)  Observations of home and facility deliveries | IDIs, PO  Thematic analysis | Women and their families were faced with many barriers to seek care when problems arose during delivery, including: (1) long and expensive referral from village facilities to higher facilities; (2) many additional costs associated with facility delivery; (3) medicine not provided by the facility and difficult to procure; (4) difficulty collecting money on short notice leads to delays - reliance on neighbors, high-interest rate loans, mortgaging land; and (5) high cost of providing food and accommodation to birth accompaniers. | High quality |
| 12 | Griffiths P & Stephenson R (2001) | Maharashtra, India | 45 women | IDI  Content analysis, thematic analysis | A woman's perception of health care was an important factor in the decision to deliver at home or at a facility. Some women valued the safe and high quality care provided at private facilities, while other women only valued facility delivery in the presence of complications. Women who delivered at home believed that TBAs provided quality care and were more comfortable delivering with them than using a facility. These women believed that facilities were intolerant of traditional birth practices and valued the cultural norm of delivering at home. Far distance from rural areas to facilities impeded a woman's ability to reach a facility, but was not as influential for women from urban areas. Women complained of a lack of transportation, especially at night. High costs associated with facility delivery was a barrier for both urban and rural women, and women tended to choose the least expensive facility, even if this compromised the quality of care. | Medium quality |
| 13 | Story W; Burgard S; Lori J; Taleb F; Ali N; Hoque D (2012) | Netrokona district, Bangladesh | 40 husbands and wives interviewed, but analysis limited to 20 husbands | IDI  Thematic analysis | Common themes of access and utilization of delivery care across husbands, regardless of delivery location, include: (1) poor transportation options and high cost of transportation; (2) direct and indirect costs associated with facility delivery; (3) awareness of delivery complications; and (4) men were responsible for instrumental support, including organizing transportation to a facility or calling a TBA. Men whose wives utilized skilled delivery care expressed emotional involvement with their wives. Men whose wives delivered with an unskilled provider believed that following traditional delivery practices was important, whereas men whose wives delivered at a facility valued the modern technology at the facility. | Low quality |
| 14 | Tabatabaie M; Moudi Z; Vedadhir A (2012) | Zahedan, Sistan and Baluchestan provinces, Iran | 38 participants total in qualitative component (21 mothers, 14 midwives, 3 TBAs) | Mixed-methods design; IDI  Grounded theory, thematic analysis | When complications arose during home birth, delays in seeking EmOC occurred for the following reasons: (1) high cost of facility delivery and insufficient insurance options; (2) fear of caesarean section; (3) lack of personal birth attendants at a facility; (4) belief in supernatural causes of disease; (5) fear of offending a TBA and disgrace in the community; and (6) lack of cooperation between midwife and physicians. | High quality |
| 15 | Gebrehiwot T; Goicolea I; Edin K; Sebastian M (2012) | Tigray province, Ethiopia | 6 FGDs with 51 women total | FGD  Grounded theory, constant comparison | Women recognized the risks associated with child birth and made pragmatic choices on their delivery location. Factors that affected delivery location included: (1) birth viewed as a natural event that can occur at home until complications arise; (2) elderly women, TBAs, and husbands play important roles in decision-making process; (3) cultural norms encourage home delivery; (4) belief that God is responsible for their birth outcomes; (5) belief that delivery at home is convenient and familiar; (6) perceived low quality of care at the facility, but perceived high efficacy of facility services; (7) transportation barriers, particularly at night; and (8) HEWs encourage facility delivery. | Medium quality |
| 16 | Turan J; Hatcher A; Medema-Wijnveen; Onono; Miller S; Bukusi E; Turan B; Cohen C (2012) | Nyanza province, Kenya | 48 participants total in qualitative component (16 women, 12 TBAs, 12 male partners or other family members, 8 CHWs) | Mixed-methods design; IDI  Thematic analysis | High prevalence of HIV in the community and HIV stigma acted as barriers to facility delivery, in conjunction with: (1) high cost of facility delivery and payment required at the time of service; (2) fear of poor treatment of women of low-SES in the facility; (3) husband's involvement in the decision to seek care; (4) perception that if a woman did not seek ANC, she could not deliver at a facility; (5) facility delivery perceived as only necessary during complications; and (6) fear of violence or verbal abuse resulting from HIV diagnosis or disclosure. | Medium quality |
| 17 | Mwangome F; Holding P; Songola K; Bomu G (2012) | Msambweni district, Kenya | 186 participants in total: 24 women in IDIs, 72 hospital staff in 6 FGDs, 36 men and 54 women in 6 FGDs | IDI, FGD  Thematic analysis | Barriers to the utilization of facilities for delivery include: (1) high cost of facility delivery; (2) poor access to hospital; (3) lack of supportive birth attendance at facility; (4) unfamiliar birth practices at facility; (5) perceived low quality of care and abuse at the facility; (6) fear of caesarean section; (7) belief that facility is only appropriate for complicated deliveries; (8) fear of HIV testing; and (9) role of other family members in decision-making. | Low quality |
| 18 | Bedford J; Gandhi M; Admassu M; Girma A (2012) | South Wollo, Ethiopia | 46 women | IDI  Grounded theory, thematic analysis | Factors that influence a woman's delivery location include: (1) perception that birth is a natural event that does not warrant medical attention; (2) acceptance of facility during complications; (3) adherence to traditional practice of home birth; (4) lack of advanced planning for child birth; (5) lack of supportive attendance at a facility; (6) far distance to a facility and lack of transportation access; (7) fear of referral; (8) collective decision-making in a household with husbands playing dominant role; and (9) ANC providers not emphasizing facility delivery. | High quality |
| 19 | Izugbara C; Kabiru C; Zulu E (2009) | Nairobi, Kenya | 74 women total in 12 FGDs | FGD  Thematic analysis | Factors affecting choice of delivery location include: (1) perception that facilities are more qualified to handle deliveries than TBAs, although TBAs have a "gift from God"; (2) perception that facility delivery is only necessary when faced with complications; (3) belief that ANC visits ensure and promote a safe delivery; (4) previous birth experiences inform future decisions; (5) high direct and indirect costs associated with facility delivery; (6) disrespect and abuse from health workers; and (7) fear of mandatory HIV testing at a facility. | Low quality |
| 20 | Osubor K; Fatusi A; Chiwuzie J (2006) | Ologbo, Nigeria | 6 FGDs conducted with approximately 8 women per FGD | Mixed-methods design;  FGD  Thematic analysis | Complications during delivery were unable to be managed in the local health center, and both TBAs and health workers felt comfortable referring women to the district facility. However, the relationship between TBAs and health workers was strained, with both sides believing that the other was uncooperative. Factors that affected care-seeking behavior among women included: (1) some complications viewed as spiritual conditions that cannot be treated at a facility (i.e.: convulsions during labor); (2) preference for TBAs; (3) perception that government facilities are undesirable locations for delivery; (4) fear of operations at a facility; and (5) husbands play a significant role in decision-making. | Low quality |
| 21 | Seljeskog L; Sundby J; Chimango J (2006) | Mangochi-Lungwena, Malawi | IDIs with 10 participants total (6 women, 2 health workers, 2 TBAs)  Unspecified number of FGDs  Unspecified number of delivery observations | IDI, FGD, PO  Content analysis, thematic analysis | The most important factors influencing the choice of delivery fall into three categories: (1) quality of care [ANC providers not encouraging facility delivery, health workers overburdened and unavailable; TBAs trusted by women, but not integrated into the health system]; (2) cultural factors [perception that facilities are safe and preferred location to ensure positive delivery outcome; decision-making influenced family, fear of caesarean section]; and (3) availability of services [far distance and inaccessible transportation to the facility and high direct and indirect costs at facility]. | Low quality |
| 22 | Turan J; Miller S; Bukusi E; Sande J; Cohen C (2008) | Kisumu, Kenya | IDIs conducted with 38 participants total (14 women, 4 male partners, 18 health workers, 2 TBAs)  Delivery observations conducted with 22 women of varying HIV status | IDI, delivery observation  Thematic analysis | Factors affecting a woman's decision to deliver at home include: (1) high cost of facility delivery; (2) lack of transportation at night & far distance to facility; (3) preference for TBAs due to traditional practices, comfort at home, devoted attention of the TBA, flexible payment, and intergenerational continuity; and (4) fear of facility delivery [poor treatment and abuse by health workers especially if HIV+, caesarean section, male health workers, HIV testing, unwanted disclosure of HIV status and wrongly-labeled as HIV+]. | High quality |
| 23 | Pembe A; Urassa D; Darj E; Carlstedt A; Olsson P (2008) | Rufiji district, Tanzania | 96 participants in total (85 community members in 8 FGDs and 11 health workers in 2 FGDs) | FGD  Content analysis, thematic analysis | Mother-in-laws are heavily involved in the care-seeking during pregnancy. The husband is responsible for raising money for a referral from other family members and friends and he has the final decision on whether the woman will attend a facility. Referrals made during ANC visits are not taken as seriously as referrals made during delivery or complications. Previous pregnancy experiences influence actions taken during the current pregnancy. The cost of travel, availability of transportation, and cost of services are barriers to accessing care. Facilities were perceived as higher quality care than a health center, but health workers often mistreated women and did not listen to them or explain procedures. | Low quality |
| 24 | Spangler S & Bloom S (2010) | Kilombero and Ulanga Districts, Tanzania | 48 IDIs with women  Unspecified number of participant observation of women, family members, health staff, and TBAs. | Mixed-methods design;  IDI, PO  Thematic analysis | The qualitative findings suggest that younger women with more education prefer facility delivery as part of their modern identity, whereas older women with less education prefer home delivery to maintain tradition. Women of higher SES tended to view childbirth as dangerous and believed that facilities would assist them and that TBAs were not quality providers. However, women of higher SES may still face issues in physically accessing a facility or may lack decision-making power. In contrast, women of lower SES faced issues of high cost of services and transportation at a facility. They feared poor treatment at the facility due to their social class and were not confident that the facility would help them during a complication. However, some women of low SES used facilities, particularly when they had a relative who worked there, a referral from ANC, lived in close proximity to a facility, or had family support. | High quality |
| 25 | Doctor H; Findley S; Ager A; Cometto G; Afenyadu G; Adamu F; Green C (2012) | Katsina, Yobe, & Zamfara states, Nigeria | 119 IDIs in total (women, community leaders, TBAs, traditional healers, & health workers)  95 FGDs in total with ~ 9 participants per FGD (women and men) | Mixed-methods design; IDI, FGD  Thematic analysis | Women viewed childbirth as a normal and natural process that did not require facility services. Women preferred to deliver at home where they would have privacy, unlike at a facility. Women who delivered at home tended to have little or no connection to the biomedical health system. Facilities were far distances away from the study communities and women had trouble accessing transportation, particularly at night. | Medium quality |
| 26 | Oyerinde K; Harding Y; Philip A; Garbrah-Aidoo N; Kanu R; Oulare M; Shoo R; Daoh K (2012) | Sierra Leone | 123 IDIs with women  20 FGDs with 160 participants in total (women, men, and TBAs) | IDI, FGD  Thematic analysis, grounded theory | Several factors impacted a woman's ability to access and utilize emergency obstetric care: (1) high cost of services; (2) inaccessible and unaffordable transportation; (3) lack of ambulance service; (4) perception that God controls birth outcomes; (5) tradition of home delivery; (6) decision-making lies with husband or mother-in-laws; (7) lack of autonomy, especially for younger unwed women; (8) fear of mistreatment and by and lack of trust of health workers; (9) delays in the transition from unskilled to skilled care; (10) long wait times in the facility; (11 perception that facilities can manage complications; (12) inadequate staffing at the facility; and (13) lack of privacy or separate maternity ward at the facility. | Medium quality |
| 27 | Sorensen B; Nielsen B; Rasch V; Elsass P (2011) | Kagera Region, Tanzania | 97 IDI participants total (31 women, 32 relatives, 19 health workers, 15 TBAs) | IDI, birth observation  Thematic analysis, informed by the actantial model | Factors that influenced a woman's use of delivery services include: (1) many actors in the decision-making process; (2) perceived low quality of care from TBAs; (3) high perceived efficacy of facility services; (4) lack of social support at a facility; (5) far distance and lack of accessible transportation to a facility, especially at night; and (6) inadequate staffing at the facility. Users recommended strengthening village health facilities and providers recommended behavior change communications in the community to improve maternal health. | Medium quality |
| 28 | Shiferaw S; Spigt M; Godefrooij M; Melkamu Y; Tekie M (2013) | Kembata Tembaro,Ethiopia | 8 IDIs in total (6 health providers and 2 TBAs)  - 3 FGDs with 23 participants in total (women, men, CHWs) | Mixed-methods design; IDI, FGD  Thematic analysis | Barriers to facility delivery in this setting included: (1) belief that TBAs are culturally acceptable and competent; (2) low cost of TBA services; (3) home birth allowed support from family during delivery; (4) perception that health workers at the facility are incompetent; (5) lack of out-of-hours availability at the facility; (6) lack of privacy at the facility; (7) perception that skilled attendance at birth is unnecessary; (8) many actors involved in decision-making regarding delivery location; (9) referrals perceived as expensive; and (10) high indirect costs of facility delivery. | Low quality |
| 29 | Øxnevad, M (2011) | Oromia, Ethiopia | 31 IDIs in total (women, elderly women, husbands, TBA, health workers)  2 FGDs with 9 women in total | IDI, FGD  Systematic text condensation, thematic analysis | Home was considered the natural place to deliver unless a complication arose, but many actors played a role in determining the location of delivery. Women were skeptical of facility deliveries because they disliked the exams and feared operations. At the facility, privacy was not guaranteed and women perceived that the health workers did not care about their well-being. Referrals for comprehensive emergency obstetric care were wrought with transportation and cost barriers. Care from TBAs was sought first, and facility delivery would be sought only if complications arose. | Medium quality |
| 30 | Parkhurst J; Rahman S; Ssengooba F (2007) | Hoima, Uganda and Jhenaidah, Bangladesh | 60 women in total (30 from Uganda and 30 from Bangladesh) | IDI  Thematic analysis | In Uganda, most women planned in advance to deliver at a facility due to a fear of childbirth risks, and husbands were responsible for organizing funds and transportation. In Bangladesh, most women planned to deliver at home and would only attend a facility in the case of a complication. Families did not anticipate or prepare for delivery costs. Several actors played a role in the decision-making process in both countries. In Bangladesh, traditional providers often had links to facilities, which encouraged women to attend a facility if she had a complication. In both countries, transportation was a barrier to facility delivery and women feared the time away from domestic responsibilities. | Low quality |
| 31 | Duong D, Binns C, Lee A (2004) | Thanh Hoa Province, Vietnam | 16 IDIs in total (women, providers, and TBAs)  -16 FGDs in total with 6-8 participants per FGD (women, mothers/mothers-in-law, & husbands/partners) | Mixed-methods design;  IDI, FGD  Thematic analysis | Factors that influenced the location of delivery included: (1) perceived high quality of care at the facility, despite a perceived limitation of technical capacity of health workers; (2) perceived high cost of facility delivery, but cost is acceptable to save a life; (3) TBAs perceived as credible practitioners; (4) poor experiences with ANC may facilitate home delivery; (5) older women influence choice of delivery location; (6) stigma against single mothers; (7) coercive population policy facilitates home birth for women with more than 2 children; and (8) birth perceived as a natural event. | Low quality |
| 32 | Mrisho M, Schellenberg JA, Mushi AK, Obrist B, Mshinda H, Tanner M, Schellenberg D (2007) | Nachingwea, Lindi, Ruangwa, Tandahimba and Newala Districts, Tanzania | 32 IDIs with women  2 FGDs in total with 6-8 women each | Mixed-methods design;  IDI, FGD, PO  Thematic analysis | Factors that influenced the location of delivery included: (1) high cost of facility delivery; (2) lack of transportation and far distance to a facility; (3) perceived poor staff attitudes; (4) lack of privacy in the facility; and (5) many actors involved in the decision-making process. | Low quality |
| 33 | Moyer C; Adongo P; Aborigo R; Hodgson A; Engmann C; Devries R (2013) | Kassena-Nankana district, Ghana | 72 IDIs in total (35 women, 13 providers, 4 TBAs, 4 herbalists, 16 community leaders)  Unspecified number of FGDs conducted with 81 grandmothers, 22 compound heads, and 78 household heads | IDI, FGD  Thematic analysis | Facility delivery is becoming more acceptable among women, particularly because of the belief that home delivery is risky. Many women viewed facility delivery as "modern" and home delivery was more likely to occur when a woman's family practiced traditional religions. Many actors played a role in the decision-making process, which often led to a delay in seeking care. Women of low-SES often felt shame if they attended a facility due to their low-SES. | High quality |
| 34 | Magoma M, Requejo J, Campbell OM, Cousens S, Filippi V (2010) | Ngorongoro, Tanzania | 13 IDIs with 11 participants (4 women, 2 husbands, 2 traditional leaders, 1 TBA, 1 elder male, 1 MCH coordinator)  15 FGDs with a total of 160 participants (women, healthcare providers, TBAs, and elders) | IDI, FGD  Thematic analysis | TBAs played an important role in the Maasai community, but were only used by the Watemi community in case of an emergency when facility delivery could not be accessed. In the Maasai community, the TBAs may dissuade women from delivering at a facility and women usually sought care from TBAs first. Women in the Watemi community tended to deliver at home with the support of female relatives and neighbors. Transport and lack of planning for a facility birth were the main barriers to facility delivery. Facility deliveries were subsidized, so cost was not an issue. Women did not believe that ANC providers were consistently counseling them about the importance of facility deliveries, and women attended ANC for reassurance that they were safe to deliver at home. Women disliked facilities due to the vaginal exams, unfamiliar birth positions, and a fear of being cut. Due to poor treatment from health workers, women preferred TBAs to accompany their deliveries. | High quality |
