# Supplementary figures and images for: Facilitators and barriers to facility-based delivery in low- and middle-income countries: a qualitative evidence synthesis
Source: Reprod Health. 2014 Sep 19;11:71. doi: 10.1186/1742-4755-11-71 (PMC4247708; doi:10.1186/1742-4755-11-71)

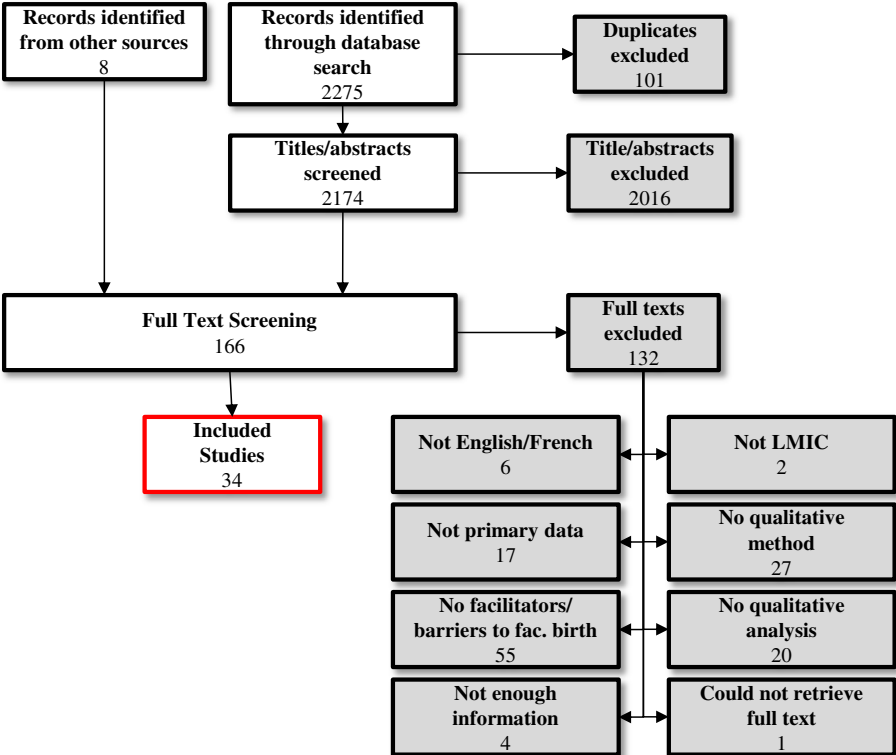

Supplement: Supplementary file 2 — Authors’ original file for figure 1 [file 12978_2014_330_MOESM2_ESM.pdf]

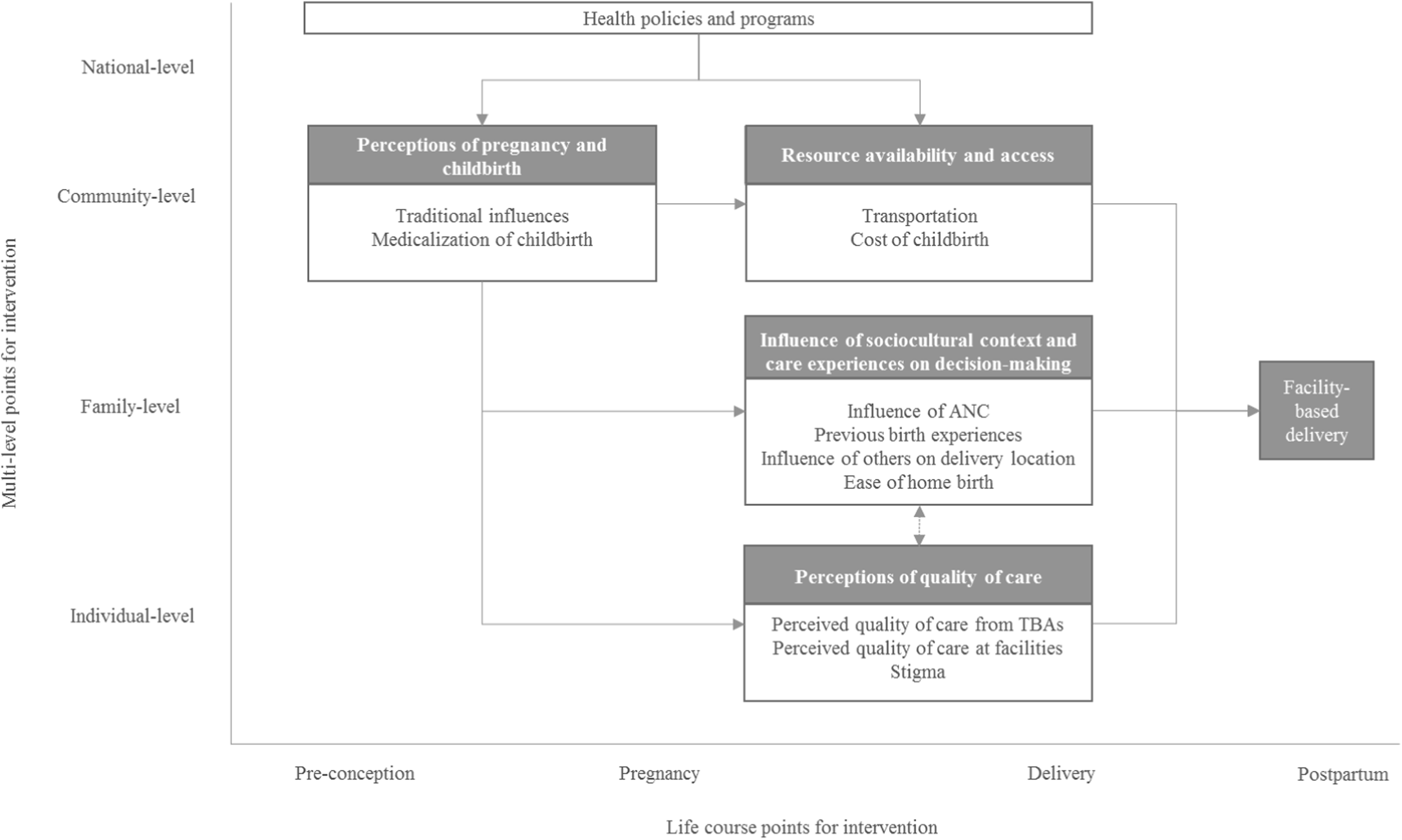

Supplement: Supplementary file 3 — Authors’ original file for figure 2 [file 12978_2014_330_MOESM3_ESM.tiff]
