# Supplementary material for: Facilitators and barriers to facility-based delivery in low- and middle-income countries: a qualitative evidence synthesis
Source: Reprod Health. 2014 Sep 19;11:71. doi: 10.1186/1742-4755-11-71 (PMC4247708; doi:10.1186/1742-4755-11-71)
Supplement: Supplementary file 4 — Authors’ original file for figure 3 [file 12978_2014_330_MOESM4_ESM.docx]

**Table 1. Thematic analysis**

| **Third order** | **Second order** | **First order** | **References** |
| --- | --- | --- | --- |
| **Perceptions of pregnancy and delivery** | **Traditional influences** | Barrier: Tradition supports an external locus of control | (14-18) |
|  |  | Barrier: Traditional understandings of disease etiology | (13,14,17-19,21) |
|  | **Medicalization of childbirth** | Barrier: Facilities deemed unnecessary for the “natural event” of birth | (16,17,22-35) |
|  |  | Facilitator: Facility delivery valued for obstetric complications | (16,17,24,27-36) |
|  |  | Barrier: Unfamiliar and undesirable birth practices in facilities | (16,17,19,22,24,27-29,34,37,38) |
|  |  | Barrier: Lack of privacy in a facility | (20,22,24,25,29,37,39) |
|  |  | Barrier: Lack of supportive attendance during facility delivery | (20-22,27,32,34,37,40) |
|  |  | Barrier: Fear of cutting | (17,19,21,22,34,40-42) |
|  |  | Facilitator: Desire for modernity | (14,16,18,22,23) |
|  |  | Barrier: Making logistical plans for childbirth is rare | (16,17,23,27,29,30) |
| **Influence of sociocultural context and care experiences** | **Influence of ANC** | Barrier: Belief that ANC diminishes the likelihood of a complicated delivery | (17,28,43) |
|  |  | Barrier: ANC providers do not universally promote facility delivery | (17,27,33,42) |
|  |  | Barrier: Lack of ANC attendance inhibits facility delivery | (25,26,29) |
|  | **Previous birth experiences** | Facilitator/barrier: Effects of previous birth experiences on subsequent delivery locations | (13,15,18,22,23,28,30,32,34,37,41,43,44) |
|  | **Influence of others on delivery location** | Barrier: Too many people involved in the decision-making process leads to delays in seeking care | (14,16,21,22,24,27,30,31,34,38,42,44) |
|  |  | Barrier: Intergenerational continuity and the role of elder women | (13-17,32,37,40,42) |
|  |  | Facilitator/barrier: The role of husbands | (13-20,22,23,26,27,29,37,44) |
|  |  | Facilitator: Personal links to healthcare facilities | (18,23,30,41) |
|  | **Ease of home birth** | Barrier: Facility births less convenient than home births | (16,31,33,39,43) |
|  |  | Barrier: Unable to maintain household or family demands during facility delivery | (13,16,17,30,31,42) |
|  | **Effects of policies** | Facilitator/barrier: Health insurance schemes, national population policies, and national policies aimed to shift deliveries from the home to a facility | (13,21,29,31,32,43,45) |

| **Resource availability and access** | **Transportation** | Barrier: Poor proximity and access to a facility | (16,18,20,23-25,27,30-34,37,42,43,46) |
| --- | --- | --- | --- |
|  |  | Barrier: Lack of accessible and reliable transportation | (20,23-25,30,34,37,42,44) |
|  |  | Barrier: Inaccessibility of transportation and facilities during off-hours | (20,31,37,40,42,43) |
|  |  | Barrier: Delays in accessing referral services | (15,27,32,42,46) |
|  | **Cost of childbirth** | Barrier: Perceived high cost of facility birth compared to home birth | (15,18,20-22,24,26,28,30-35,37,39,43,45,46) |
|  |  | Barrier: Lack of access to funds in an emergency | (20,26,30-32,35,45,46) |
|  |  | Barrier: Indirect and hidden costs associated with facility delivery | (14,17,18,20,22,23,28,29,32,40-42,44-46) |
| **Perceptions of quality of care** | **Perceived quality of care from TBAs** | Barrier: Utilization of TBAs as first-line providers | (16,21,22,28,30,31,35,37,38,40-43) |
|  |  | Facilitator: TBAs perceived as providing low quality care | (18,28,31,37,40,42) |
|  |  | Barrier: TBAs perceived as providing high quality care | (13,16,17,19,28,31,33,38,42) |
|  | **Perceived quality of care at facilities** | Facilitator: Facilities perceived as providing high quality care | (13-17,20,22,27,28,31-33,37,42,44) |
|  |  | Barrier: Facilities perceived as providing low quality of care | (15-17,21,24,27-29,34,37) |
|  |  | Barrier: Mistreatment and abuse by health workers | (13,15-18,20,22,26,32,34,35,39,42-45) |
|  |  | Barrier: Neglect and delays in receiving care at the facility | (15,20,22,29,34,37,42,43,45) |
|  |  | Barrier: Inadequate health facility staffing and infrastructure | (15,16,20,22,32,33,35,37,42-44) |
|  | **Stigma** | Barrier: Fear of compulsory HIV testing during delivery services | (26,28,34,36) |
|  |  | Barrier: Fear of HIV-status disclosure in health facilities | (26,34,36) |
|  |  | Barrier: Fear of treatment disparities among HIV-positive women | (26,36) |
|  |  | Barrier: Stigmatization of unwed, pregnant women | (13,15,20) |
