# Supplementary material for: Facilitators and barriers to facility-based delivery in low- and middle-income countries: a qualitative evidence synthesis
Source: Reprod Health. 2014 Sep 19;11:71. doi: 10.1186/1742-4755-11-71 (PMC4247708; doi:10.1186/1742-4755-11-71)
Supplement: Supplementary file 5 — Authors’ original file for figure 4 [file 12978_2014_330_MOESM5_ESM.docx]

**Table 2: Summary of findings**

| **#** | | **Factors that affect the utilization of facility-based deliveries** | | **Relevant papers** | | **Confidence in the evidence** | | **Explanation of confidence in the evidence assessment** | |
| --- | --- | --- | --- | --- | --- | --- | --- | --- | --- |
| 1 | | **Barrier: Tradition supports an external locus of control**  Across sub-Saharan Africa, religious faith and traditional religious practices played a role in decision-making regarding delivery location. Wwomen described their trust in God and the belief that God controls their destiny. These traditional beliefs contributed to a sense of fatalism as some women believed that delivery complications were beyond their control. | | (24-28) | | Moderate confidence | | In general, the studies were moderately well done. The finding was seen across several studies and settings. | |
| 2 | | **Barrier: Traditional understandings of disease etiology**  Seeking care at medical facilities may have been delayed in situations when women or their families viewed certain health problems as spiritual rather than physical in nature, influenced by their traditional understandings of disease etiologies. | | (23,24,27-29,31) | | Moderate confidence | | In general, the studies were moderately well done. The finding was seen across several studies and settings. | |
| 3 | | **Barrier: Facilities deemed unnecessary for the “natural event” of birth**  The perception that birth is a natural life event rather than a medical procedure emerged as a common theme in many of the primary studies across a variety of contexts. Respondents therefore saw no rationale for delivering at a facility, and paying to do so was considered illogical and superfluous. | | (26,27,32-45) | | High confidence | | In general, the studies were moderately well done. The finding was seen across many studies and settings. | |
| 4 | | **Facilitator: Facility delivery valued for obstetric complications**  Many women across different contexts attempted home delivery first and considered facilities acceptable only if complications arose during the delivery process. Although facility-based delivery was not the first choice for many women, they acknowledged the importance of facilities in cases of complicated birth. | | (26,27,34,37-46) | | High confidence | | In general, the studies were moderately well done. The finding was seen across many studies and settings. | |
| 5 | | **Barrier: Unfamiliar and undesirable birth practices in facilities**  When faced with the prospect of facility birth, some women may fear unfamiliar or undesirable procedures, such as unfamiliar birthing positions and intrusive vaginal exams. Hospital providers were sometimes perceived to conduct too many digital vaginal examinations, which women found uncomfortable and dehumanizing. Some women also preferred delivering at home with a TBA because they had more control over their birth position than delivering at a facility. | | (26,27,29,32,34,37-39,44,47,48) | | High confidence | | In general, the studies were moderately well done. The finding was seen across many studies and settings. | |
| 6 | | **Barrier: Lack of privacy in a facility**  Many women felt that they had more control over maintaining their privacy when delivering at home compared to the facility. Privacy is greatly valued by parturient women, yet it may not be well-maintained in a facility due to a lack of cultural sensitivity and dismissive attitudes towards poor women, coupled with the lack of private labor wards. | | (30,32,34,35,39,47,49) | | Moderate confidence | | In general, studies were moderately well done. The finding was seen across several studies and settings. | |
| 7 | | **Barrier: Lack of supportive attendance during facility delivery**  One of the most salient differences between home birth and facility birth was the perceived lack of supportive attendance at birth in a facility. Women commonly referred to their families and TBAs as providing supportive and comforting care, and receiving physical, social, and emotional support from their family during delivery was vitally important for the parturient woman. Facility policies limiting the involvement of TBAs and family members during birth induced anxiety in many women. | | (30-32,37,42,44,47,50) | | Moderate confidence | | In general, studies were moderately well done. The finding was seen across several studies and settings. | |
| 8 | | **Barrier: Fear of cutting**  Across multiple contexts, women referred to a “fear of cutting” as a deterrent to pursuing facility delivery. Women who mentioned a fear of cutting usually did not differentiate between episiotomy and a caesarean section; rather, they referred to any form of perineal or abdominal incision as “cutting”. Women feared cutting due to perceived longer hospital stays, higher cost, perceived unjustified operation, social stigma, and potential problems with future sexual relations. | | (27,29,31,32,44,50-52) | | Moderate confidence | | In general, studies were moderately well done. The finding was seen across several studies and settings. | |
| 9 | | **Facilitator: Desire for modernity**  Despite the role of tradition in delivery practices, women, husbands, and traditional leaders commented on changing societal norms regarding the location of delivery. In some contexts, women viewed facility delivery as a modern or contemporary idea and as something to which they aspire. | | (24,26,28,32,33) | | Moderate confidence | | In general, studies were moderately well done. However, this finding was only seen in 4 countries. | |
| 10 | | **Barrier: Making logistical plans for childbirth is rare**  Across several contexts, the lack of planning in advance for childbirth, including the decision about location of delivery, transportation planning, and acquiring liquid assets to pay for associated childbirth costs, prevented women from accessing facility delivery. Families often lack the resources to develop coping mechanisms for future events. Therefore, the capacity to make plans in low-resource households is inherently difficult. Women and their families viewed childbirth as an unpredictable event, which made creating a birth plan difficult. | | (26,27,33,37,39,40) | | Moderate confidence | | In general, studies were moderately well done. The finding was seen across several studies and settings. | |
| 11 | | **Barrier: Belief that ANC diminishes the likelihood of a complicated delivery**  Some women viewed ANC as a means to ensure a normal pregnancy and childbirth and to prepare for *home* delivery. A facility delivery would therefore not be considered unless an ultrasound during an ANC visit suggested that the mother or baby were in danger because ultrasounds are believed to be able to predict whether or not a woman will have an uncomplicated or “normal” delivery. Furthermore, ANC itself was understood by some to actually reduce the risk of complications during delivery, which may help to explain why in some contexts ANC coverage is near universal while facility delivery rates remain low. | | (27,38,53) | | Low confidence | | In general, studies were moderately well done. However, this finding was only present in 3 studies in 3 countries. | |
| 12 | | **Barrier: ANC providers do not universally promote facility delivery**  ANC providers may not be adequately advising women of the importance of facility-based care during delivery. Providers may also neglect to discuss the importance of planning ahead, instead only suggesting facility-based delivery for women with identifiable danger signs. ANC providers may be unintentionally encouraging home births by providing information on making home-birth safer (i.e. providing advice on safe home-based cord cutting measures), thus validating the practice. | | (27,37,43,52) | | Low confidence | | In general, studies were moderately well done. However, this finding was only present in 4 studies in 4 countries. | |
| 13 | | **Barrier: Lack of ANC attendance inhibits facility delivery**  Some women may not feel comfortable delivering in a facility if they have not attended ANC, even if they otherwise desire a facility birth. These women may fear mistreatment from heath workers for not possessing an ANC card or may avoid the facility due to poor experiences during ANC care. | | (35,36,39) | | Low confidence | | In general, studies were moderately well done. However, this finding was only present in 3 studies in 3 countries. | |
| 14 | | **Facilitator and barrier: Effects of previous birth experiences on subsequent delivery location**  Across a variety of contexts, women determined their level of risk for complicated deliveries based on their prior delivery experiences and birth outcomes, and these previous birth experiences may act as either a facilitator or barrier to future delivery deliveries. In many contexts, a woman’s first delivery is considered the riskiest since she has no prior experiences with child birth. Women who had previous cesarean sections or obstetric complications may desire future facility delivery due to higher perceived risk. However, if a woman gave birth to her first child without complications, utilizing a facility for subsequent births may be viewed as unnecessary or illogical. Likewise, previous negative experiences with facility births may deter women from delivering at a facility during a future birth. | | (23,25,28,32,33,38,40,42,44,47,51,53,54) | | High confidence | | In general, studies were moderately well done. Diverse findings were seen across many studies and settings. | |
| 15 | | **Barrier: Too many people involved in the decision-making process leads to delays in seeking care**  Across many contexts, parturient women may not be in full control of the decision to seek facility-based delivery, instead relying on the decisions made by many actors, including elder women, husbands, family members, and neighbors. These actors may have competing interests in the choice of a woman’s delivery location, and obtaining advice and approval from them often delays or prevents facility delivery, particularly because these decisions are often sought after labor has begun. | | (24,26,31,32,34,37,40,41,44,48,52,54) | | High confidence | | In general, the studies were moderately well done. The finding was seen across many studies and settings. | |
| 16 | | **Barrier: Intergenerational continuity and the role of elder women**  Across a variety of contexts, elder women, including mothers, mothers-in-law and grandmothers of parturient women, hold the greatest influence and decision-making power regarding delivery location. Some women believed that they should choose the same delivery location that their mothers and grandmothers experienced, in order to maintain their identity and intergenerational continuity. Other women may be pressured by the elder women to deliver at home. | | (23-27,42,47,50,52) | | High confidence | | In general, the studies were moderately well done. The finding was seen across many studies and settings. | |
| 17 | | **Facilitator and barrier: The role of husbands**  The husband plays a complex role in facilitating or preventing his wife from accessing facility-based delivery and this role varies across different contexts. In some settings, a husband may act as a facilitator by persuading his wife to visit a facility and mobilizing the necessary transportation and funds. In contrast, a husband may prohibit a facility visit altogether due to financial or cultural constraints. In other settings, the husband may play a more neutral role and place the decision to seek care in someone else’s hands, such as elder female family members. Although this finding was explored in 15 studies across 9 countries, the role of husbands varied so greatly both within and between study populations that it is difficult to draw any macro-level conclusions other than that the husband plays an important role in deciding where to deliver. | | (23-30,32,33,36,37,39,47,54) | | Low confidence | | In general, the studies were moderately well done. The role of the husband was seen across many studies and settings. However, the diverse range of roles that husbands play makes it difficult to draw conclusions on whether their role is a facilitating or inhibiting factor in accessing facility delivery. | |
| 18 | | **Facilitator: Personal links to healthcare facilities**  Families with social connections to skilled providers may be more accepting of the biomedical approach to maternity care and thus more willing to seek a facility-based delivery. More importantly, a relative or friend working at a nearby facility can often arrange quicker admission or quality treatment of a parturient woman. However, this finding was only seen in 4 studies across 3 countries, including 3 studies in Bangladesh. | | (28,33,40,51) | | Low confidence | | In general, studies were moderately well done. However, the finding was only from 3 countries. | |
| 19 | | **Barrier: Facility births less convenient than home births**  In several contexts, women preferred to deliver at home, where they were in a familiar and convenient setting. During a homebirth, a woman would not need to arrange for child care or transportation, could rest in her own bed after delivery, and be catered to by her family and friends. | | (26,41,43,49,53) | | Moderate confidence | | In general, studies were moderately well done. The finding was seen across several studies and settings. | |
| 20 | | **Barrier: Unable to maintain household or family demands during facility delivery**  Some women felt that they could exert greater control on their domestic responsibilities when they delivered at home and were concerned that their domestic responsibilities, such as child care, cooking, cleaning, gardening and tending the livestock, would be abandoned if they attended a health facility for delivery. | | (23,26,27,40,41,52) | | Moderate confidence | | In general, studies were moderately well done. The finding was seen across several studies and settings. | |
| 21 | | **Barrier: Poor proximity and access to a facility**  Geographical distance to a health facility is an influential factor affecting a woman’s delivery location, explored in 16 studies across 11 countries. Women residing in both urban and rural areas where health services do not exist at the community level may face considerable traveling time to reach a facility. The perceived far distance to health facilities may create a dependency on home birth as some women report that the facility is too far to travel to during labor, particularly given the restricted transportation options. | | (26,28,30,33-35,37,40-44,47,52,53,56) | | High confidence | | In general, the studies were moderately well done. The finding was seen across many studies and settings. | |
| 22 | | **Barrier: Lack of accessible and reliable transportation**  Poor availability of transportation played a crucial role in the decision to deliver at a facility and whether or not it could be reached in a timely manner. In the absence of a reliable private car, women were faced with arduous modes of transportation including bicycle, rickshaw, motorcycle, boat, walking, or public transportation, which was often intermittent in rural areas. | | (30,33-35,40,44,47,52,54) | | Moderate confidence | | In general, studies were moderately well done. The finding was seen across several studies and settings. | |
| 23 | | **Barrier: Inaccessibility of transportation and facilities during off-hours**  Travel at night or on weekends was considered particularly difficult as there are fewer public transportation options, women may be afraid of thieves and wild animals, and the price is higher. Even if women are able to arrange transportation during the off-hours, health facilities may be closed or lack the staffing to manage her delivery. | | (30,41,47,50,52,53) | | Low confidence | | In general, studies were of low quality. The finding was seen across several studies and settings. | |
| 24 | | **Barrier: Delays in accessing referral services**  Organizing referrals for obstetric complications was a time-consuming and arduous process, complicated by a lack of access to transportation, good roads, adequate funds, and communication systems. The lack of coordination between different health system actors also contributed to delays in reaching care. | | (25,37,42,52,56) | | Moderate confidence | | In general, studies were moderately well done. The finding was seen across several studies and settings. | |
| 25 | | **Barrier: Perceived high cost of facility birth compared to home birth**  Direct costs associated with childbirth were perceived to be unaffordable for many women and some women perceived themselves as too poor to deliver in a facility. Where women viewed childbirth as a non-medical event, the cost of childbirth is considered extraneous and unnecessary. This finding was explored in 19 studies across 12 countries. | | (25,28,30-32,34,36,38,40-45,47,49,53,55,56) | | High confidence | | In general, the studies were moderately well done. The finding was seen across many studies and settings. | |
| 26 | | **Barrier: Lack of access to funds in an emergency**  Low-SES families often did not plan in advance for costs associated with child birth and few families had assets or savings to devote to health expenses, thus causing a scramble to raise funds during obstetric complications. Collecting the necessary money was a difficult task as few banks or moneylenders would lend money to the poor, and if they did, exorbitant interest rates could make the principle escalate rapidly in just a few months. Instead, family members were often sent around the community to collect money from their neighbors or try to sell property or livestock | | (30,36,40-42,45,55,56) | | Moderate confidence | | In general, studies were moderately well done. The finding was seen across several studies and settings. | |
| 27 | | **Barrier: Indirect and hidden costs associated with facility delivery**  Even in settings where direct delivery costs were subsidized, families were expected to pay for transportation to the facility, drugs, medical supplies (i.e.: gloves, needles, gauze), blood for transfusions, laboratory services, food during the hospital stay, bribes to health providers, and laundry services. These additional costs often came as a surprise to women after they attended the facility, which may impact their future choice of delivery location. In addition to the extra point-of-care costs associated with facility birth, families experienced opportunity costs due to absence from work and domestic responsibilities. | | (24,27,28,30,32,33,38,39,42,50-52,54-56) | | High confidence | | In general, the studies were moderately well done. The finding was seen across many studies and settings, but predominantly in Bangladesh and Tanzania. | |
| 28 | | **Barrier: Utilization of TBAs as first-line providers**  TBAs played an important role as first-line providers for many women and this role was discussed in 13 studies across 10 countries. Women emphasized the close bond that they felt with TBAs, due to their status in the community and the trust they developed over years of experience. This relationship often prompted women to desire home-based births attended to by a TBA rather than a facility. | | (26,31,32,38,40,41,45,47,48,50-53) | | High confidence | | In general, studies were moderately well done. The finding was seen across many studies and settings. | |
| 29 | | **Facilitator: TBAs perceived as providing low quality care**  Despite the bond that many women had with TBAs in their community, some women perceived TBAs as providers of low quality delivery care. These women did not trust the TBAs’ skills, knowledge, or ability to handle complications and may be more likely to seek facility-based delivery. | | (28,38,41,47,50,52) | | Moderate confidence | | In general, the studies were moderately well done. The finding was seen across several studies and settings. | |
| 30 | | **Barrier: TBAs perceived as providing high quality care**  Other women perceived TBAs as providing high quality delivery care, often emphasizing the supportive and emotional role that TBAs play. These women may believe that TBAs have innate skills gifted to them from God and that TBAs are more dependable providers than facility-based health workers. | | (23,26,27,29,38,41,43,48,52) | | Moderate confidence | | In general, the studies were moderately well done. The finding was seen across many studies and settings. | |
| **Experiences with facility providers** | | | | | | | | | |
| 31 | | **Facilitator: Facilities perceived as providing high quality care**  In contexts where facilities are perceived as providing high quality care, women may seek facility delivery to ensure positive birth outcomes. They may view facilities as providing efficacious and respectable care, and health workers as compassionate experts. It is important to note that within the same study area, perceptions of facility-based care vary greatly and participants more commonly perceived facilities to have low quality of care than high quality of care. However, women who perceived facilities as providing high quality care reportedly felt more comfortable seeking facility-based delivery. | | (23-27,30,32,37,38,41-43,47,52,54) | | High confidence | | In general, the studies were moderately well done. The finding was seen across many studies and settings. | |
| 32 | | **Barrier: Facilities perceived as providing low quality of care**  Across multiple contexts, the failure of health workers to manage severe obstetric complications contributed to a negative image of facility delivery. Women may lack confidence in the abilities of the health workers, who they consider to be undertrained, lacking skills, incompetent, inexperienced, and offering inaccurate diagnoses. It is important to note that even within the same study area, perceptions of facility-based care vary greatly. However, women who perceived facilities as providing low quality care reportedly felt less likely to seek facility-based delivery. | | (25-27,31,34,37-39,44,47) | | High confidence | | In general, the studies were moderately well done. The finding was seen across many studies and settings. | |
| 33 | | **Barrier: Mistreatment and abuse by health workers**  Many women referred to poor patient-provider interactions as a barrier to seeking delivery care. Women described providers as verbally abusive, rude, bossy, unhelpful, disrespectful, critical, easily angered, having a poor attitude, and lacking compassion. Respondents reported that facility-based providers shout at, physically abuse, and insult women during delivery. | | (23,25-28,30,32,36,42,44,45,49,52-55) | | High confidence | | In general, the studies were moderately well done. The finding was seen across many studies and settings. | |
| 34 | | **Barrier: Neglect and delays in receiving care at the facility**  Upon arrival to a facility, women often experienced delays in care provision and health workers were often slow to respond to patient needs. Health workers often did not communicate with the woman or her family on the progress of labor. | | (25,30,32,39,44,47,52,53,55) | | Moderate confidence | | In general, the studies were moderately well done. The finding was seen across several studies and settings. | |
| 35 | | **Barrier: Inadequate health facility staffing and infrastructure**  Inadequate staffing and infrastructure in the facilities contributed to the perceived low quality of care. The lack of adequate staffing led to overburdened lower-level providers and often prompted women to visit untrained traditional providers to respond to the gaps in service. | | (25,26,30,32,42,43,45,47,52-54) | | Moderate confidence | | In general, the studies were moderately well done. The finding was seen across many studies and settings. | |
| **Experiences with stigmatization in facilities** | | | | | | | | | |
| 36 | | **Barrier: Fear of compulsory HIV testing during delivery services**  In high HIV prevalence settings, a fear of compulsory HIV testing during facility-based delivery sometimes prompted women to avoid facilities altogether. These women feared the shock, stress, and depression caused by a positive HIV test, often believing that knowledge of one’s own positive HIV-status was as equally deleterious as the virus itself. This finding was present in 4 studies in 1 country (Kenya), so the certainty of the finding across multiple contexts is low, but may be higher in the Kenyan context. | | (36,38,44,46) | | Low confidence | | In general, the studies were moderately well done. However, the finding was only from 4 studies in Kenya. Therefore, the confidence of the finding across multiple contexts is low, but may be higher in Kenya. | |
| 37 | | **Barrier: Fear of HIV-status disclosure in health facilities**  Women feared unwanted disclosure of their positive HIV-status in a facility, which could lead to tremendous social, psychological, physical, and economic consequences. Crowded maternity wards, public administration of ARVs, and health workers’ failure to maintain strict confidentiality sometimes caused women to avoid facility deliveries. Again, this finding was present in 3 studies in 1 country (Kenya), so the certainty of the finding across multiple contexts is low, but may be higher in the Kenyan context. | | (36,44,46) | | Low confidence | | In general, the studies were moderately well done. However, the finding was only from 3 studies in Kenya and may only be applicable to high HIV prevalence settings. | |
| 38 | | **Barrier: Fear of treatment disparities among HIV-positive women**  Some HIV-positive women may be provided with lower quality of care due to health workers’ fear of HIV infection. However, this finding was only present in 2 studies in 1 country (Kenya). | | (36,46) | | Low confidence | | In general, the studies were moderately well done. However, the finding was only from 2 studies in Kenya. | |
| 39 | | **Barrier: Stigmatization of unwed, pregnant women**  Most societies view pregnancy and childbirth as the outcome of a marital relationship, thereby potentially stigmatizing and disempowering unwed women seeking facility delivery. Delivering at home was a desirable choice for unwed women or adolescents to avoid embarrassment or discrimination at a facility, particularly because these women were often lacking emotional and financial support from their partner or parents. However, this finding was only present in 3 studies in 3 countries (Sierra Leone, Tanzania, and Vietnam). | | (23,25,30) | | Low confidence | | In general, the studies were moderately well done. However, the finding was only from 3 studies in Sierra Leone, Tanzania, and Vietnam. | |
